# Network analysis retrieving bioactive compounds from Spirulina (*Arthrospira platensis*) and their targets related to systemic lupus erythematosus

**DOI:** 10.1371/journal.pone.0309303

**Published:** 2024-08-29

**Authors:** Amnart Chaiprasert, Ping Han, Teeraphan Laomettachit, Marasri Ruengjitchatchawalya

**Affiliations:** 1 Biotechnology Program, School of Bioresources and Technology (SBT), King Mongkut’s University of Technology Thonburi (KMUTT), Bang Khun Thian, Bangkok, Thailand; 2 Department of Medicine, Phramongkutklao Hospital, Bangkok, Thailand; 3 Bioinformatics and Systems Biology Program, SBT, KMUTT, Bang Khun Thian, Bangkok, Thailand; 4 Bioinformatics and Systems Biology Program, School of Information Technology, KMUTT, Bang Mod, Bangkok, Thailand; 5 Algal Biotechnology Research Group, Pilot Plant Development and Training Institute (PDTI), KMUTT, Bang Khun Thian, Bangkok, Thailand; Instituto Tecnologico de Monterrey, MEXICO

## Abstract

Immunosuppressive drugs are essential for systemic lupus erythematosus (SLE) treatment, but there are concerns about their toxicity. In this study, *Arthrospira platensis* was used as a resource for screening of the SLE-related bioactive compounds. To discover the potential compounds, a total of 833 compounds of *A*. *platensis* C1 were retrieved from the Spirulina-Proteome Repository (SpirPro) database and by literature mining. We retrieved structures and bioassays of these compounds from PubChem database; and collected approved and potential drugs for SLE treatment from DrugBank and other databases. The result demonstrated that cytidine, desthiobiotin, agmatine, and anthranilic acid, from the alga, has Tanimoto matching scores of 100% with the following drugs: β-arabinosylcytosine/cytarabine, d-dethiobiotin, agmatine, and anthranilic acid, respectively. The bioassay matching and disease-gene-drug-compound network analysis, using VisANT 4.0 and Cytoscape, revealed 471 SLE-related genes. Among the SLE-related genes, MDM2, TP53, and JAK2 were identified as targets of cytarabine, while PPARG and IL1B were identified as targets of d-dethiobiotin. Binding affinity between the drug ligands and the algal bioactive compound ligands with their corresponding receptors were similarly comparable scores and stable, examined by molecular docking and molecular dynamic simulations, respectively.

## Introduction

Spirulina (*Arthrospira platensis*) is a cyanobacterium that is used as a dietary supplement. Spirulina contains high amounts of proteins, vitamins, and a lot of important bioactive compounds [1]. Beneficial effects of Spirulina supplement have been studied in many medical conditions, e.g. controlling blood glucose levels and improving the lipid profile of subjects with type 2 diabetes mellitus [2]. Spirulina has been reported to enhance immune responses against viral infections such as HIV [3] and chronic hepatitis C [4], and to exhibit anti-inflammatory effects by dose-dependently inhibiting mast cell-mediated immediate-type allergic reactions *in vivo* and *in vitro* [5, 6].

In humans, Spirulina enhances both the mucosal and systemic immune systems. Its immunomodulatory activity and benefits have been demonstrated in patients with allergic rhinitis [7] and elderly people [8]. The alga also demonstrated immunosuppressive effects on both humoral and cell-mediated immune responses [9]. Despite these beneficial effects, not much is known regarding the responsible bioactive compounds and their mechanisms of action. Two main immunologic pathways in humans, namely the B-cell and T-cell receptor pathways, have been implicated in the development of systemic lupus erythematosus (SLE) [10]—a progressive autoimmune disease with unknown etiology, which can virtually affect any organ of the human body. Immunologic abnormalities, especially the production of several antinuclear antibodies, are another prominent feature of the disease. Women, especially in their 20s and 30s, are affected more frequently than men. B-cells are central to the expression of the disease. In addition to producing autoantibodies, which mediates tissue damage, B-cells process and present antigens and autoantigens to T-cells and contribute to disease expression. SLE patients with a major glomerular inflammation, lupus nephritis, are usually treated with many immunosuppressive drugs, e.g. corticosteroids, cyclophosphamide, azathioprine, mycophenolate, mammalian target of rapamycin inhibitor (mTORi), and calcineurin inhibitor [11]; however, there are concerns relating to their toxicity. Research has strived to improve the effectiveness of potential drugs and limit their side effects. The promising role of Spirulina in humans with immunologic conditions has been demonstrated [12, 13]. Understanding drug targets in a complex cellular network is crucial in selecting the proper treatment for SLE. Insight into such mechanisms may facilitate the development of combination therapies, with the aim of achieving higher efficacy and reducing the side effects of immunosuppressive drugs.

This study aims to identify Spirulina (*A*. *platensis* C1) bioactive compounds that are related to SLE immunologic response and treatment using structural similarity, bioassay similarity, disease-gene-drug-compound network analysis, molecular docking, and molecular dynamic (MD) simulation.

## Materials and methods

### Data and tools

Metabolic pathways and compounds of Spirulina were retrieved from the organism-specific database of *A*. *platensis* C1, Spirulina-Proteome Repository (SpirPro; http://spirpro.sbi.kmutt.ac.th/) database [14] and Kyoto Encyclopedia of Genes and Genomes (KEGG; www.genome.jp/kegg/) database. Additional compounds were also retrieved by literature mining using text mining. Duplicates were removed from the data to get the non-redundant compounds. All potential SLE treatment agents were searched using the terms: “lupus”, “systemic lupus erythematosus”, “SLE”, as recommended by NCBI (Medical Subject Heading; MeSH) and the International Statistical Classification of Diseases and Related Health Problems, 10th revision (ICD-10). All the approved and potential agents for SLE treatment were collected from the various databases, namely DrugBank (http://www.drugbank.ca/), KEGG, PubChem (https://pubchem.ncbi.nlm.nih.gov/), Therapeutic Targets Database (TTD; http://bidd.nus.edu.sg/group/ttd/ttd.asp), DailyMed (http://dailymed.nlm.nih.gov/dailymed/about.cfmt/), Anatomical Therapeutic Chemical (ATC) classification system and the Defined Daily Dose (DDD) system. Structural similarity matching between the compounds retrieved from *A*. *platensis* C1 and all the potential agents for SLE treatment were analyzed using the tool in PubChem.

Bioassays associated with SLE disease were searched in PubChem (http://www.ncbi.nlm.nih.gov/pcassay/?term=systemic+lupus+erythematosus). A total of 660 SLE assay identifiers (AIDs) were retrieved. Bioassay similarity matching between AIDs of compounds from *A*. *platensis* C1 and 660 AIDs of SLE was analyzed.

**Fig 1:**
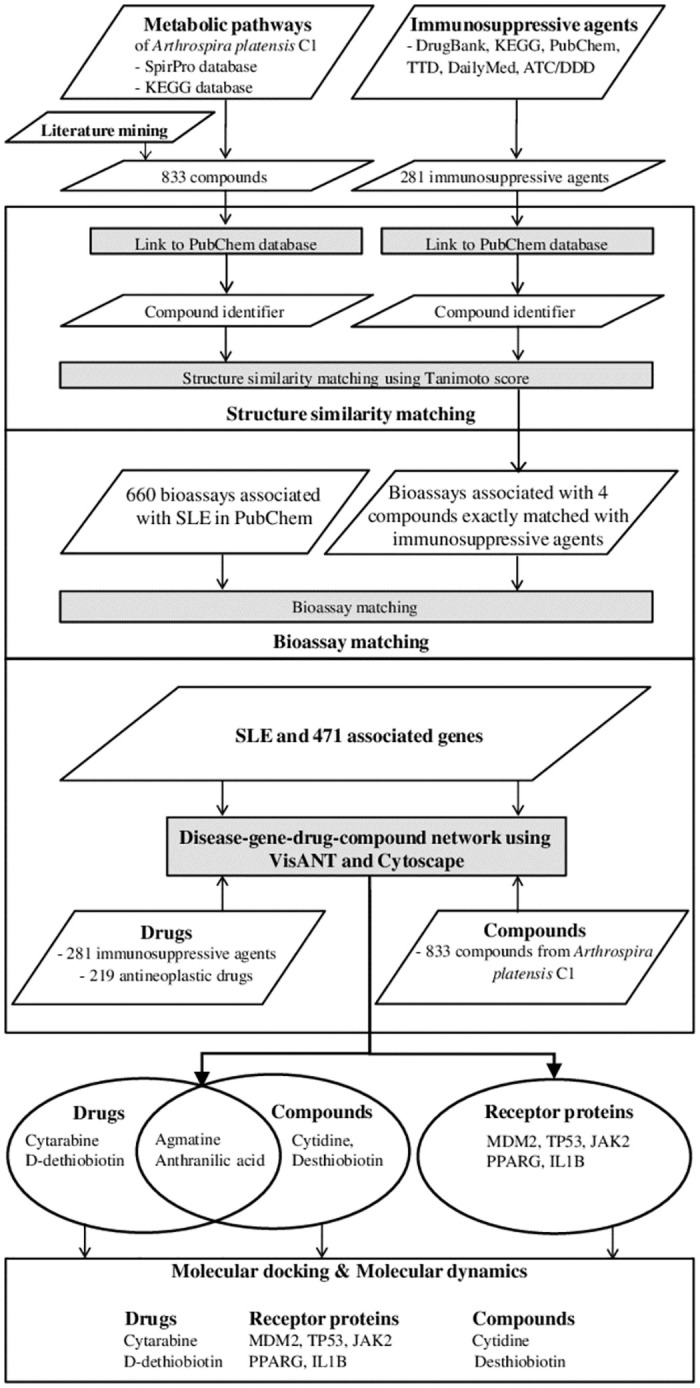
Workflow to discover potential bioactive compounds from *A*. *platensis* C1 for SLE treatment. The compounds and immunosuppressive agents (potential agents for SLE treatment) were retrieved from databases and literature mining. Using PubChem, all the compound numbers were identified; then, structural similarity matching between the compounds and the potential agents was analyzed, in parallel with searching for the bioassays associated with SLE disease. After that, disease-gene-drug-compound were networked using VisANT and visualized using Cytoscape. The networking results provided the potential immunologic effect compounds and their target genes/proteins, which were verified by molecular docking and molecular dynamics for their intermolecular interactions.

To create the disease-gene-drug interaction network for SLE, VisANT 4.0 (http://visant.bu.edu), an integrative network platform [15] was used. The compounds from *A*. *platensis* C1 were mapped onto the interaction network, and the disease-gene-drug-compound network was visualized using Cytoscape [16]. Integration of all these data in the network provided insights into the potential immunologic effect of *A*. *platensis* C1 compounds as well as information on their target genes/proteins (Fig 1).

Molecular docking was used to examine the binding affinity score between Spirulina compounds (ligands) and candidate targets (receptors). The goal of docking is to predict the best binding model of the ligand and receptor. MGLTools (http://mgltools.scripps.edu/) and PyRx (https://pyrx.sourceforge.io/) were used for molecular docking. In both tools, AutoDock was applied in the docking process. The 3D structure of Spirulina compounds were retrieved from the PubChem (https://pubchem.ncbi.nlm.nih.gov) and inputted into the MGLTools and PyRx, while the 3D structure of receptor proteins were retrieved from Protein Data Bank (PDB, https://www.rcsb.org) in.pdb file format. For docking using AutoDock, the ligands and receptors in.pdb file format were converted into.pdbqt file format by addition of charges. MD simulation was performed to confirm the binding stability of the compounds and receptor proteins. AMBER 16 software with ff14SB force field was used to perform MD simulation for 30 ns or until the complex reached an equilibrium. To evaluate the stability of simulated model, the root-mean-square displacement (RMSD) of protein backbone, complex, and ligand of complex were computed by PTRAJ module of AMBER.

## Results

### Compounds disclosure from *A*. *platensis* C1

A total of 95 metabolic pathways of *A*. *platensis* C1 (S1 Table) were retrieved from the SpirPro database [14]. The main metabolic pathways of this organism were related to carbohydrate, amino acid, and vitamin metabolism, which consisted of 15, 13, and 11 pathways, respectively. To find the compounds in *A*. *platensis* C1, enzymes that produced the metabolic products from substrates were firstly sought. Seventy-nine (79) of the 95 pathways revealed enzyme-related metabolites, while the other 16 pathways did not. A total of 699 EC numbers (enzyme commission numbers in the KEGG database) were retrieved (S2 Table). The syn00230 pathway associated with purine metabolism had the highest EC numbers with 41 enzymes. Many of these complex pathways contained only few Spirulina-specific EC numbers. The 415 non-redundant enzymes were collected resulting in the total production of 968 products. After removing duplicated products, the remaining 369 non-redundant products were collected. These products were linked to 369 substance identifiers (SIDs) in the PubChem database. However, based on compound identifiers (CIDs), chemical structures of only 302 compounds were available. Literature review and text mining of published data were further done to find all known and potential chemical compounds in *A*. *platensis* C1. A genome-scale metabolic model of *A*. *platensis* C1 containing 692 genes, 837 metabolites, and 875 reactions has been reported [17], from which a total of 501 compounds were retrieved with their relevant CIDs. After combining the data retrieved from SpirPro [14] and literature review, and excluding all redundancies, a total of 833 CIDs (S3 Table) were obtained and used for further analysis.

### Computational screening of immunosuppressive agents

Data for both approved and potential immunosuppressive drugs for SLE treatment were retrieved from the databases (DrugBank, KEGG, PubChem, TTD, DailyMed, and the ATC/DDD). These drugs and their identify numbers were matched with CID numbers in the PubChem database. Proteins and chemical compounds related to immunologic effects were also searched in PubChem database, using the terms “immunomodulator”, “immunosuppression”, “immunosuppressive”, and “immunosuppressive agents” as recommended by NCBI (Medical Subject Heading; MeSH). The corresponding GenInfo Identifier (GI) numbers were also collected. After the data were combined and redundancies excluded, a total of 281 chemical compounds related to immunologic effects were obtained (S4 Table). These were later matched with the compounds retrieved from *A*. *platensis* C1.

### Structural similarity matching between compounds retrieved from *A*. *platensis* C1 and immunosuppressive agents

To find the algal potential bioactive compounds to be used as SLE drugs or immunosuppressive agents, matching between the potential compounds retrieved from *A*. *platensis* C1 and immunosuppressive agents was performed. Details of the 234,073 similarity matchings with their scores are shown (S1 Fig). The structural similarity matching between 833 algal compounds and 281 immunosuppressive agents results in 1637, 412, 203, 103, 28, and 4 matchings with Tanimoto scores of ≥ 70%, ≥ 80%, ≥ 85%, ≥ 90%, ≥ 95%, and 100%, respectively (S5 Table). Four compounds; cytidine, desthiobiotin, agmatine, and anthranilic acid, demonstrate 100% matching with known immunosuppressive agents (Table 1), whereas others such as metrotrexate had a matching score of 87% with folic acid. In addition, lobenzarit had a matching score of 80% with anthranillic acid, 72% with 4- aminobenzoic acid, and 71% with 1-(2-carboxyphenylamino)-1-deoxyribulose 5-phosphate.

**Table 1:** Example of *A*. *platensis* C1 compounds and immunosuppressive agents with structural similarity scores ≥ 70%.

Compound	Immunosuppressive agent	Similarity score (%)
Cytidine	β-arabinosylcytosine	100
	Cytarabine	100
	Arabinofuranosylcytosine (Iretin)	100
Desthiobiotin	Desthiobiotin	100
	D-dethiobiotin	100
Agmatine	Agmatine	100
Anthranilic acid	Anthranilic acid	100
	Lobenzarit	80
Folic acid	Metrotrexate	87
4-Aminobenzoic acid	Lobenzarit	72
1-(2-carboxyphenylamino)-1-deoxyribulose 5-phosphate	Lobenzarit	71

Furthermore, there were 103 matchings with Tanimoto scores of ≥ 90%. The immunosuppressive agents, namely 8-aminoguanosine, 3868-32-4 (2,8-Diamino-9-[3,4-dihydroxy-5-(hydroxymethyl)-2-oxolanyl]-3H-purin-6-one), fludarabine phosphate, and cladribine had the highest matching frequencies of 24 (23.3%), 24 (23.3%), 16 (15.5%), and 11 (10.7%), respectively. The compounds: cytidine, 5’-cytidylic acid, cytidine 5’-diphosphate, desthiobiotin, and cytidine triphosphate exhibit lower matching frequency 8 (7.8%), 6 (5.8%), 6 (5.8%), 3 (2.9%), and 3 (2.9%), respectively.

### Bioassay matching between compound bioassay of *A*. *platensis* C1 and bioassay associated with SLE

All proteins and bioassays associated with SLE were searched in PubChem database. A total of 660 bioassays and their relevant assay identifier (AID) numbers were retrieved (S6 Table). Four compounds and their related bioassays were matched with the 660 bioassays associated with SLE. AIDs and targets (proteins or nucleotides) of these matchings between bioassays associated with SLE and bioassays associated with immunosuppressive compounds, were as follows; SLE and cytidine: AID504734 protein target-inhibitor of human Toll-like receptor 9; SLE and anthranilic acid: AID651758 protein target-interleukin 8 stimulation. The SLE matching bioassays described above were all non-active. However, cytidine, a nucleoside molecule, has 7 active bioassays with antibacterial and anticancer activities against *Plasmodium falciparum* and Ewing’s sarcoma, respectively. Whereas desthiobiotin has only 1 active bioassay based on *in vivo* anticancer drug screening in tumor model induced by Friend leukemia virus in mice. Though, cytidine and desthiobiotin were respectively identified in the pyrimidine and biotin metabolic pathways in *A*. *platensis*, the latter has been defined as an immunosuppressive agent in MESH term (https://www.ncbi.nlm.nih.gov/mesh/67004749). Most of the approved drugs used in the treatment of SLE and potential drugs were classified as immunosuppressant and antineoplastic. Consequently, cytidine and desthiobiotin exhibiting anticancer activities could be used as potential drug candidates in the treatment of SLE.

### Disease-gene-drug-compound network for SLE

**Fig 2:**
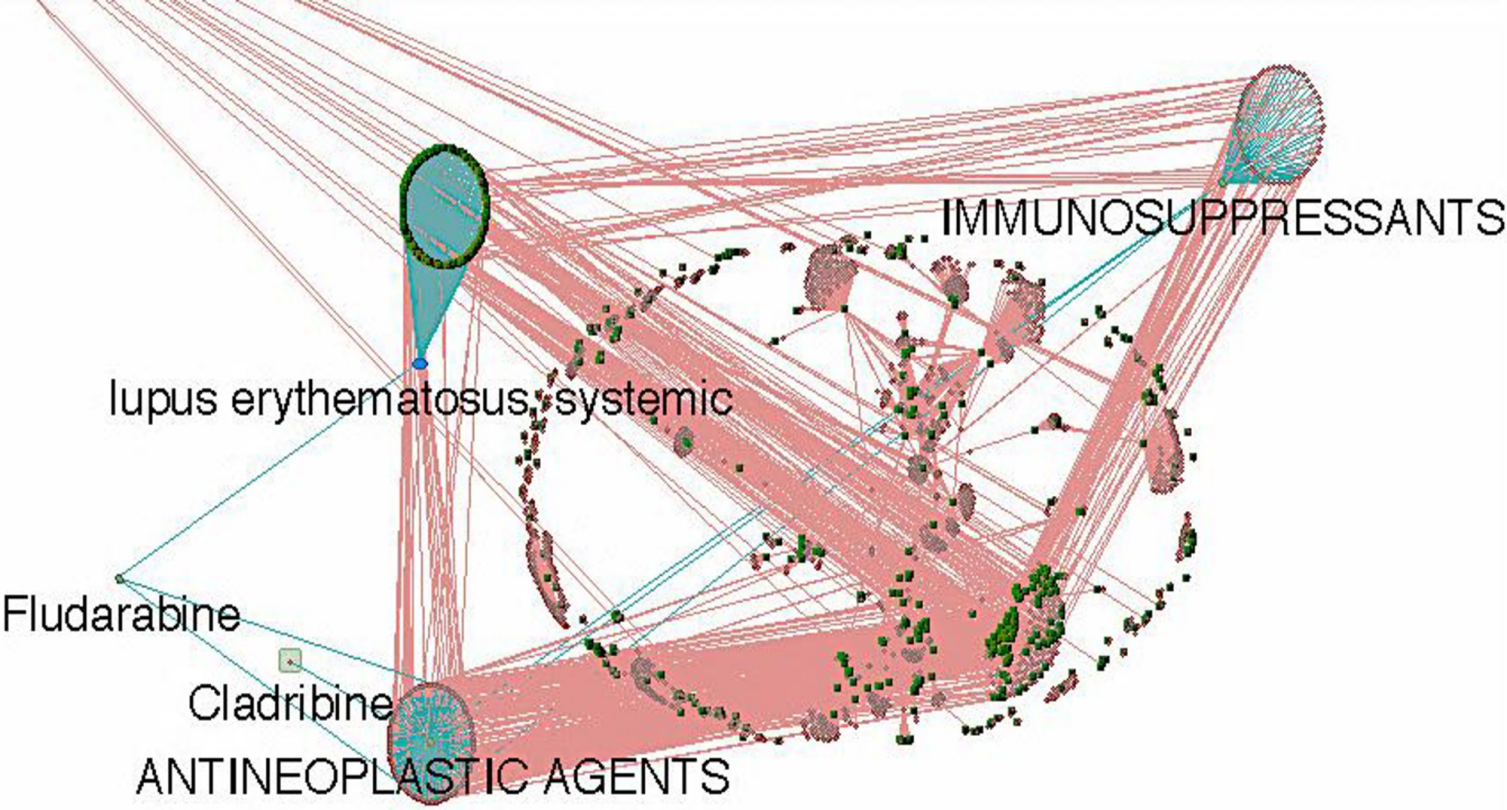
SLE disease-gene-drug network. Using VisANT 4.0 to demonstrate the association between SLE-related genes and immunosuppressive or antineoplastic agents (fludarabine phosphate and cladribin) with highly structural similarity to compounds from *A*. *platensis* C1. The network consists of 471 genes/proteins associated with SLE, 219 antineoplastic drugs, and 54 immunosuppressive drugs. Blue node; SLE disease, green node; gene/protein, red node; drug, blue edge; immunosuppressant & antineoplastic drugs and gene interactions, red edge; other drug-gene interactions.

The disease-gene-drug network was created using VisANT 4.0. The network consisted of 471 genes/proteins associated with SLE, 219 antineoplastic drugs, and 54 immunosuppressive drugs (Fig 2). From the matchings between 833 *A*. *platensis* C1 compounds and 281 immunosuppressive agents (Tanimoto scores of ≥ 90%); two immunosuppressive agents, namely fludarabine phosphate [18] and cladribine [19], were included in SLE-gene-drug network with only fludarabine phosphate found to be associated with the SLE-related genes.

**Fig 3:**
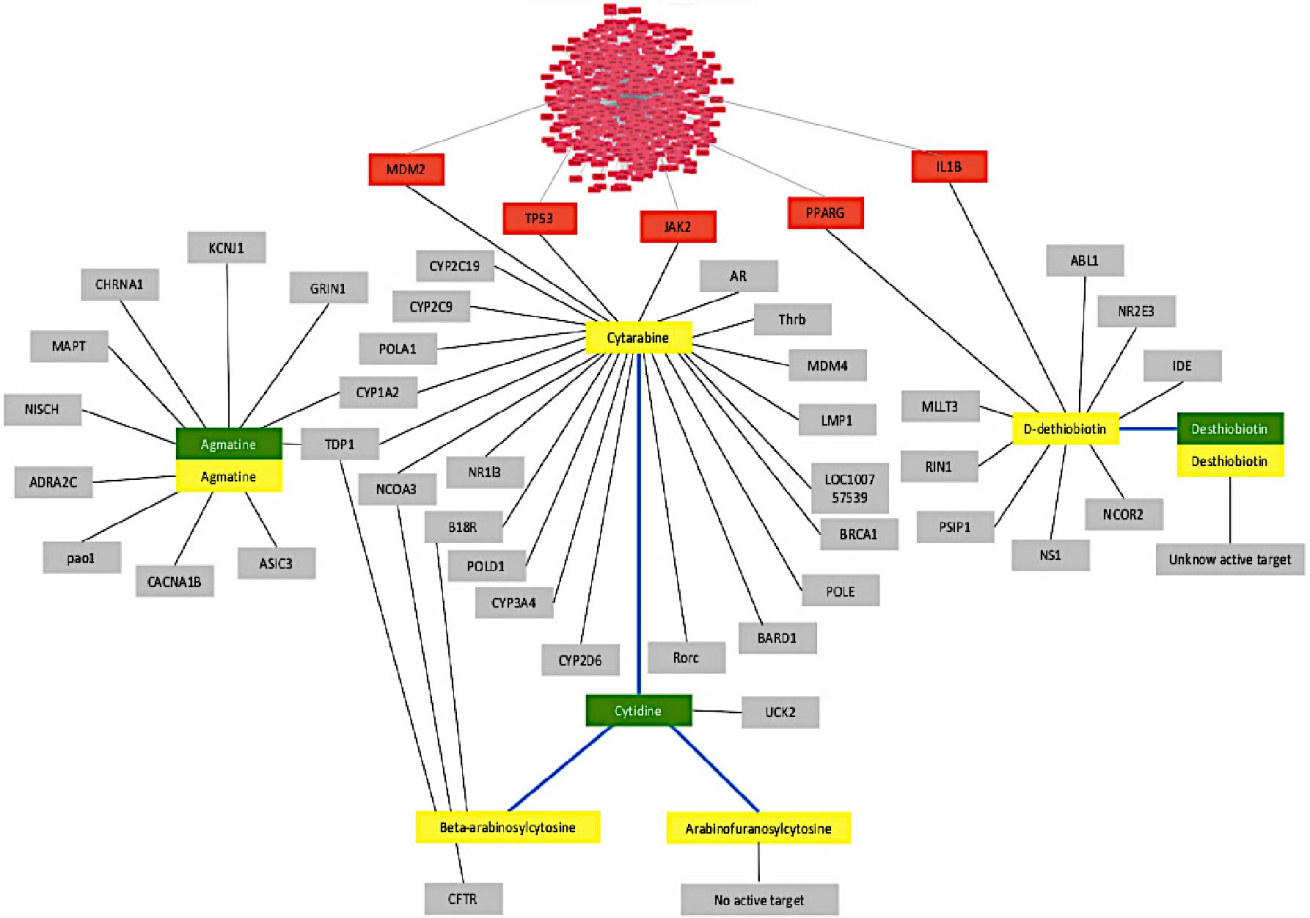
Networking of SLE-related genes, immunosuppressive agents, and bioactive compounds from *A*. *platensis* C1. The network created by Cytoscape consisted of 471 genes/proteins associated with SLE, 6 immunosuppressive agents, and 3 bioactive compounds from *A*. *platensis* C1. The structural similarity between the bioactive compound and the immunosuppressive agent is a score of 100%. Gray node; target gene, Red node; target gene associated with SLE, yellow node; immunosuppressive agent, green node; bioactive compound, blue line.

The disease-gene-drug-compound network was created by mapping 4 bioactive compounds from *A*. *platensis* C1 with the highest potential to be immunosuppressive agents, and 471 genes/proteins associated with SLE. The interaction network was visualized using Cytoscape (Fig 3). Integration of these data in the network offered insights into the potential immunologic effect of the compounds and their target genes/proteins. The target genes for cytarabine were MDM2, TP53, and JAK2, and that for d-dethiobiotin were PPARG and IL1B. These genes were identified to be associated with SLE. Cytidine and desthiobiotin, from *A*. *platensis* C1, had 100% structural similarity score with cytarabine and d-dethiobiotin, respectively.

### Molecular docking and molecular dynamic simulation of potential bioactive compounds

**Fig 4:**
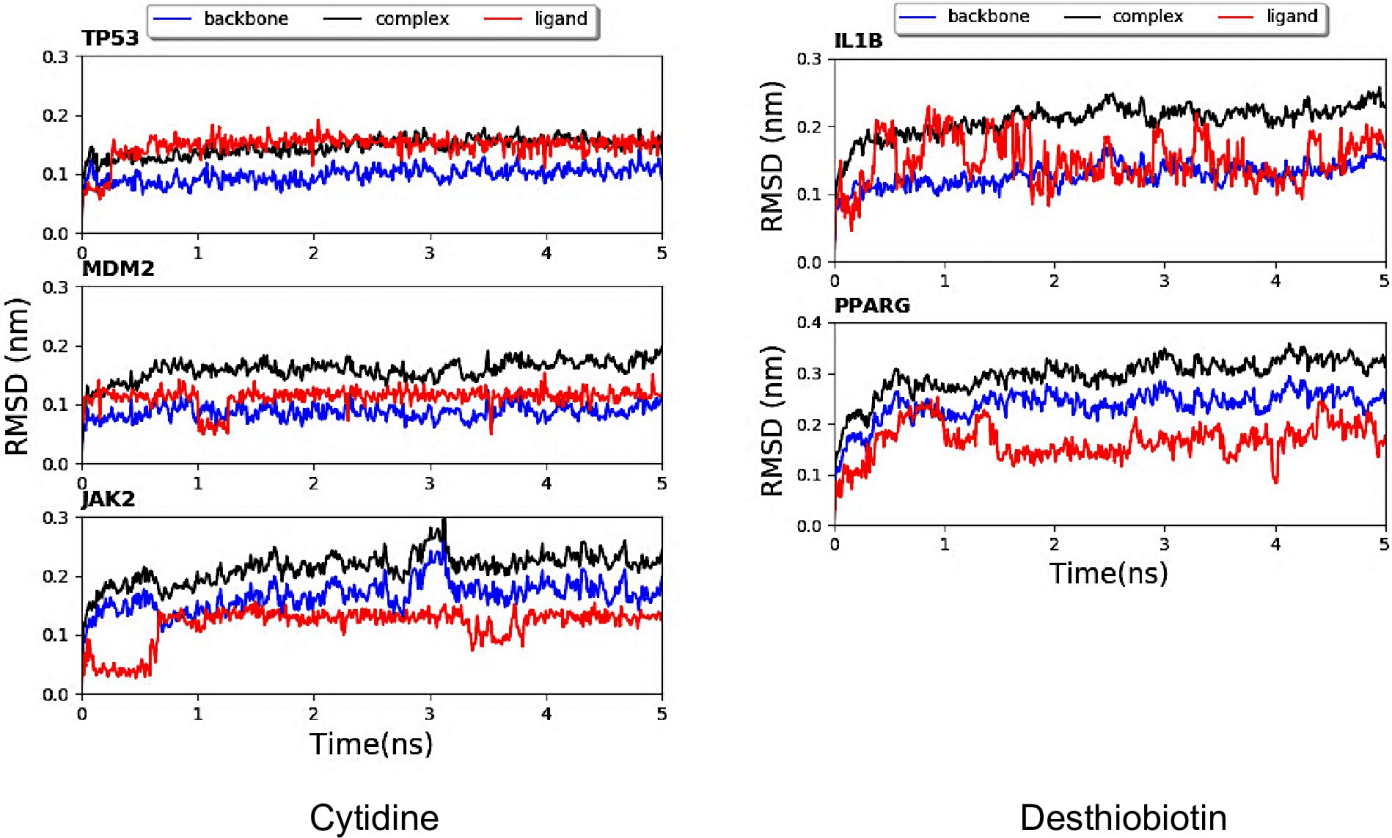
Potential drug effect demonstrated by binding affinity of the bioactive compounds from *A*. *platensis* C1 with their corresponding receptors using RMSD. Cytidine complexes with JAK2; MDM2; TP53, and Desthiobiotin complexes with PPARG; IL1B. The root-mean-square displacement (RMSD) plot for backbone (blue), complex (black), and ligand (red).

Our prior analysis indicated that cytidine, desthiobiotin, agmatine, and anthralinic acid were bioactive compounds with high propensity for SLE treatment. The first two compounds; cytidine and desthiobiotin were not annotated as immunosuppressive drugs. To predict the binding affinities between the compounds; cytarabine (drug) and cytidine (bioactive compound) and their corresponding receptors; MDM2, TP53, and JAK2, a molecular docking was performed. The binding affinity between d-dethiobiotin (drug), desthiobiotin (bioactive compound), and the receptors; PPARG and IL1B were also predicted. Both cytidine and desthiobiotin had strong binding score (low negative free energy of the binding) with their corresponding receptors, comparable to cytarabine and d-dethibiotin (Table 2). Additionally, MD simulation was performed to confirm the binding affinity and stability between the compounds and receptors. The RMSD plot for backbone, complex, as well as that of cytidine, desthiobiotin and their corresponding receptors were shown (Fig 4). The RMSD values of the complexes were rather stable and maintained within the fluctuation of 1 Å. In addition, the RMSD values of the complexes showed a very small fluctuation and reached equilibrium within 5 ns. The RMSD of backbone displayed similar fluctuation pattern with that of the complex.

**Table 2:** Potential drug effect demonstrated by the binding affinity scores between the ligands (drugs and bioactive compounds) with their corresponding receptors through molecular docking.

Receptor protein	Docking energy (kcal/mol); mean (SD)	
	Cytarabine (Drug)	Cytidine (Bioactive compound)
MDM2	-4.34 (0.40)	-4.59 (0.36)
TP53	-5.96 (0.21)	-6.12 (0.26)
JAK2	-6.01 (0.21)	-6.27 (0.32)
	**D-dethiobiotin (Drug)**	**Desthiobiotin (Bioactive compound)**
PPARG	-5.72 (0.36)	-5.4 (0.45)
IL1B	-4.93 (0.20)	-5.04 (0.24)

## Discussion

SLE is a chronic autoimmune disease with a wide spectrum of clinical features and unknown etiology [10]. Serious side effects may occur with conventional immunosuppressive treatment. Identification of biological therapeutic agents targeting molecular mediators with limited side effects can be challenging. Spirulina has immunomodulatory activity, and its benefits have been studied in patients with various diseases [7, 8, 12]. Spirulina consumption significantly improved symptoms in patients with allergic rhinitis in a double blind, placebo-controlled study [7]. Consuming 3 to 8 g of Spirulina per day for 12 to 16 weeks has favorable effects on immune variables in the elderly [8, 12]. Despite these beneficial effects, little is known about the responsible bioactive compounds and their mechanisms of action. In this study, the metabolic pathways, and potential bioactive compounds of *A*. *platensis* C1 for SLE treatment were investigated. Unlike other strains, the alga produces a single colony with non-gliding ability; and can be used for arsenic culture selection. Based on these abilities, this strain is used for genetic studies in the laboratory. Extensive research on both lipid desaturation mechanisms and physiological conditions for cell growth have been performed using this strain. The whole genome sequence of *A*. *platensis* C1 at the GenBank database of the Center for Biotechnology Information (NCBI, http://www.ncbi.nlm.nih.gov/genome/?term=Arthrospira+platensis) is 6,089,210 bp long and contains 6,108 protein-coding genes, 45 RNA genes, and no plasmids [20]. Though the metabolic pathway of *A*. *platensis* from the Encyclopedia of Genes and Genomes (KEGG) database belongs to *A*. *platensis* NIES39. Fortunately, an organism-specific database for *A*. *platensis* C1 has been created and can be retrieved from the Spirulina-Proteome Repository (SpirPro) database.

Immunosuppressive drugs are the mainstays of treatment for SLE [21]. Some antineoplastic drugs are used to treat autoimmune diseases. Drugs used to treat other autoimmune diseases may also work for SLE treatment because these conditions are characterized by an overactive immune system. In this study, all approved and potential drugs or agents for SLE treatment were searched and used for analysis. Immunosuppressive, immunomodulatory, and antineoplastic agents were collected from various databases, namely DrugBank, KEGG, PubChem, TTD, DailyMed, and ATC/DDD. The DrugBank database is a unique bioinformatics and cheminformatics resource that combines detailed drug data with comprehensive drug target information. It contains 12,147 drug entries including 2,557 approved small molecule drugs, 1,285 approved biotech (protein/peptide) drugs, 130 nutraceuticals, and over 5,865 experimental drugs. Additionally, 5,167 proteins (i.e., drug target/enzyme/transporter/carrier) sequences are linked to these drug entries. The DailyMed web contains 108,551 drug listings as submitted to the Food and Drug Administration (FDA). It provides high quality information about marketed drugs. The ATC/DDD is a tool for exchanging and comparing data on drug use at international, national, or local levels. The ATC/DDD has become the gold standard for international drug utilization research. This system is developed and maintained by the WHO Collaborating Centre for Drug Statistics Methodology, http://www.whocc.no. A total of 281 drugs and chemical compounds related to immunologic effect were collected from these databases.

From the structural similarity matchings between the compounds retrieved from *A*. *platensis* C1 and immunosuppressive agents with Tanimoto scores of ≥ 90%, fludarabine phosphate was one of the immunosuppressive agents with the most frequent matching. Its chemical structure resembles that of adenosine monophosphate found in *A*. *platensis* C1. Fludarabine phosphate was found to be associated with SLE-related genes and may be used to treat this autoimmune disease [18, 22, 23]. Adenosine monophosphate plays an important role in many cellular metabolic processes, and it is also a component in the synthesis of RNA. It is found in many kinds of organism, therefore not a unique and interesting compound in Spirulina.

Cytidine had structural similarity matching with three immunosuppressive agents: β-arabinosylcytosine, arabinofuranosylcytosine, and cytarabine, demonstrating a Tanimoto score of 100%. The target genes for cytarabine are MDM2, TP53, and JAK2, these genes are associated with SLE. Cytidine also displayed bioassay activity against Ewing’s sarcoma. The antineoplastic activity of cytidine may be used for autoimmune disease treatment. Desthiobiotin had structural similarity matching with d-dethiobiotin (an immunosuppressive agent) with Tanimoto score of 100%. The target genes for d-dethiobiotin are PPARG and IL1B; these genes are associated with SLE. Desthiobiotin has been defined as an immunosuppressive agent in MESH term. Using a mice tumor model with Friend leukemia virus, desthiobiotin demonstrated one active bioassay in an in vivo anticancer drug screening study. Cytidine and desthiobiotin are respectively identified in the pyrimidine and biotin metabolic pathways of *A*. *platensis*. Agmatine and anthranilic acid, which are found in *A*. *platensis* C1, are classified as immunosuppressive agents in various drug databases.

Our findings that identified cytidine and desthiobiotin as bioactive compounds in *A*. *platensis* C1 with the highest potential for use as immunosuppressive agents against SLE. The results from disease-gene-drug-compound network for had been confirmed by molecular docking and MD simulations. Both cytidine and desthiobiotin had good docking score with their corresponding receptors, similar to cytarabine and d-dethibiobin; the immunosuppressive agents. A relatively stable RMSD of cytidine and desthiobiotin with SLE target proteins indicated their high propensity for disease specific therapy. Agmatine and anthranilic acid may be used for SLE treatment because of their immunosuppressive activities. These bioactive compounds should be clinically studied for their medical use in relation to SLE.

Several studies have shown that Spirulina enhances both mucosal and systemic immune systems. It has immunosuppressive effects on both humoral and cell-mediated immune responses, and its immunomodulatory activity in patients with allergic rhinitis and in elderly patients are well documented. The Spirulina bioactive compounds and their mechanisms of action in these conditions and other autoimmune diseases need to be further investigated and established.

## Conclusions

We screened the SLE-related bioactive compounds from Spirulina (*A*. *platensis* C1) using multi-steps/methods and tools including data mining of the algal bioactive compounds and potential immunosuppressive agents SLE treatment; structural similarity matching and the bioassays associated with SLE disease; disease-gene-drug-compound networking, before verifying by molecular docking and molecular dynamic simulation for the intermolecular interactions of the potential compounds and their target genes/proteins. Our result demonstrated that cytidine and desthiobiotin are the algal bioactive compounds with high propensity for use as immunosuppressive agents against SLE, regards to their binding affinity and stability with their corresponding receptors: JAK2; MDM2; TP53, and PPARG; IL1B, respectively.

## Supporting information

S1 Fig Frequency distribution of the 234,073 structural similarity matchings between 833 compounds retrieved from *A*. *platensis* C1 and 281 immunosuppressive agents, with Tanimoto scores ranging from 0 to 100%. (PDF)

S1 Table List of 95 metabolic pathways of *A*. *platensis* C1 from Spirulina-Proteome Repository (SpirPro) database. The SpirPro is a web-based tools analysis of proteome data integrated with protein-protein interactions and/or at metabolic pathways from KEGG, http://spirpro.sbi.kmutt.ac.th/.
(PDF)

S2 Table List of 699 enzymes from 95 metabolic pathways of *A*. *platensis* C1 retrieved from Spirulina-Proteome Repository (SpirPro) database. SpirPro is based on proteomic data and interactome data inference from orthologous proteins in another cyanobacterium, *Synechocystis* sp. PCC 6803, and incorporates this information into KEGG pathways.
(PDF)

S3 Table List of 833 compounds retrieved from *A*. *platensis* C1, along with their corresponding compound identifier numbers in the PubChem database. (PDF)

S4 Table List of 281 agents/drugs with known immunosuppressive or immunomodulating activities, along with their corresponding compound identifier numbers in the PubChem database. (PDF)

S5 Table Examples and frequency of structural similarity matchings between 833 compounds retrieved from *A*. *platensis* C1 and 281 immunosuppressive agents with high Tanimoto scores ranging from 60 to 100%. (PDF)

S6 Table List of 660 bioassay identifier numbers associated with systemic lupus erythematosus, retrieved from the PubChem database. (PDF)

## Abbreviations

AID: assay identifiers
CID: compound identifier
EC: enzyme commission
KEGG: Kyoto Encyclopedia of Genes and Genomes
SID: substance identifier
SLE: systemic lupus erythematosus
SpirPro: Spirulina-Proteome Repository
