# Supplementary material for: Network analysis retrieving bioactive compounds from Spirulina (Arthrospira platensis) and their targets related to systemic lupus erythematosus
Source: PLoS One. 2024 Aug 29;19(8):e0309303. doi: 10.1371/journal.pone.0309303 (PMC11361558; doi:10.1371/journal.pone.0309303)

S1 Fig. Frequency distribution of the 234,073 structural similarity matchings between 833 compounds retrieved from *A. platensis* C1 and 281 immunosuppressive agents, with Tanimoto scores ranging from 0 to 100%.

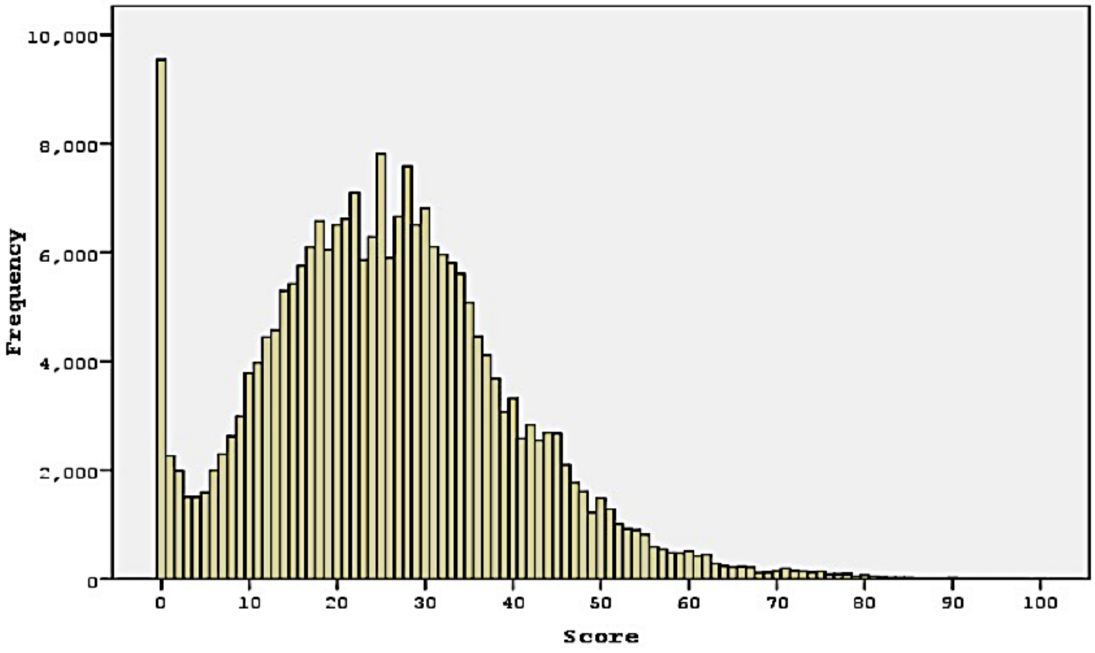

Supplement: S1 Fig — (PDF) [file pone.0309303.s001.pdf]
