# Supplementary material for: Network analysis retrieving bioactive compounds from Spirulina (Arthrospira platensis) and their targets related to systemic lupus erythematosus
Source: PLoS One. 2024 Aug 29;19(8):e0309303. doi: 10.1371/journal.pone.0309303 (PMC11361558; doi:10.1371/journal.pone.0309303)
Supplement: S1 Table — The SpirPro is a web-based tools analysis of proteome data integrated with protein-protein interactions and/or at metabolic pathways from KEGG, http://spirpro.sbi.kmutt.ac.th/. (PDF) [file pone.0309303.s002.pdf]

S1 Table. List of 95 metabolic pathways of *A. platensis* C1 from Spirulina-Proteome Repository (SpirPro) database.

The SpirPro is a web-based tools analysis of proteome data integrated with protein-protein interactions and/or at metabolic pathways from KEGG, http://spirpro.sbi.kmutt.ac.th/.

No	Pathway	Entry	Name
1	Carbo_Met	syn00010	Glycolysis / Gluconeogenesis - *Synechocystis* sp. PCC 6803
2	Carbo_Met	syn00020	Citrate cycle (TCA cycle) - *Synechocystis* sp. PCC 6803
3	Carbo_Met	syn00030	Pentose phosphate pathway - *Synechocystis* sp. PCC 6803
4	Carbo_Met	syn00040	Pentose and glucuronate interconversions - *Synechocystis* sp. PCC 6803
5	Carbo_Met	syn00051	Fructose and mannose metabolism - *Synechocystis* sp. PCC 6803
6	Carbo_Met	syn00052	Galactose metabolism - *Synechocystis* sp. PCC 6803
7	Carbo_Met	syn00053	Ascorbate and aldarate metabolism - *Synechocystis* sp. PCC 6803
8	Lipid_Met	syn00061	Fatty acid biosynthesis - *Synechocystis* sp. PCC 6803
9	Lipid_Met	syn00071	Fatty acid degradation - *Synechocystis* sp. PCC 6803
10	Lipid_Met	syn00100*	Steroid biosynthesis
11	CfVit_Met	syn00130	Ubiquinone and other terpenoid-quinone biosynthesis - *Synechocystis* sp. PCC 6803
12	Enrgy_Met	syn00190	Oxidative phosphorylation - *Synechocystis* sp. PCC 6803
13	Enrgy_Met	syn00195	Photosynthesis - *Synechocystis* sp. PCC 6803
14	Enrgy_Met	syn00196	Photosynthesis - antenna proteins - *Synechocystis* sp. PCC 6803
15	Nucle_Met	syn00230	Purine metabolism - *Synechocystis* sp. PCC 6803
16	Nucle_Met	syn00240	Pyrimidine metabolism - *Synechocystis* sp. PCC 6803
17	Amino_Met	syn00250	Alanine, aspartate and glutamate metabolism - *Synechocystis* sp. PCC 6803

18	Amino_Met	syn00260	Glycine, serine and threonine metabolism - *Synechocystis* sp. PCC 6803
19	Amino_Met	syn00270	Cysteine and methionine metabolism - *Synechocystis* sp. PCC 6803
20	Amino_Met	syn00280	Valine, leucine and isoleucine degradation - *Synechocystis* sp. PCC 6803
21	Amino_Met	syn00290	Valine, leucine and isoleucine biosynthesis - *Synechocystis* sp. PCC 6803
22	Amino_Met	syn00300	Lysine biosynthesis - *Synechocystis* sp. PCC 6803
23	Amino_Met	syn00310	Lysine degradation - *Synechocystis* sp. PCC 6803
24	Amino_Met	syn00330	Arginine and proline metabolism - *Synechocystis* sp. PCC 6803
25	Amino_Met	syn00340	Histidine metabolism - *Synechocystis* sp. PCC 6803
26	Amino_Met	syn00350	Tyrosine metabolism - *Synechocystis* sp. PCC 6803
27	Amino_Met	syn00360	Phenylalanine metabolism - *Synechocystis* sp. PCC 6803
28	XenBD_Met	syn00361	Chlorocyclohexane and chlorobenzene degradation - *Synechocystis* sp. PCC 6803
29	XenBD_Met	syn00362	Benzoate degradation - *Synechocystis* sp. PCC 6803
30	XenBD_Met	syn00363	Bisphenol degradation - *Synechocystis* sp. PCC 6803
31	XenBD_Met	syn00364	Fluorobenzoate degradation - *Synechocystis* sp. PCC 6803
32	Amino_Met	syn00380	Tryptophan metabolism - *Synechocystis* sp. PCC 6803
33	Amino_Met	syn00400	Phenylalanine, tyrosine and tryptophan biosynthesis - *Synechocystis* sp. PCC 6803
34	OSecM_BS	syn00401	Novobiocin biosynthesis - *Synechocystis* sp. PCC 6803
35	OAmin_Met	syn00410	beta-Alanine metabolism - *Synechocystis* sp. PCC 6803
36	OAmin_Met	syn00430	Taurine and hypotaurine metabolism - *Synechocystis* sp. PCC 6803
37	OAmin_Met	syn00450	Selenocompound metabolism - *Synechocystis* sp. PCC 6803

38	OAmin_Met	syn00460	Cyanoamino acid metabolism - *Synechocystis* sp. PCC 6803
39	OAmin_Met	syn00471	D-Glutamine and D-glutamate metabolism - *Synechocystis* sp. PCC 6803
40	OAmin_Met	syn00473	D-Alanine metabolism - *Synechocystis* sp. PCC 6803
41	OAmin_Met	syn00480	Glutathione metabolism - *Synechocystis* sp. PCC 6803
42	Carbo_Met	syn00500	Starch and sucrose metabolism - *Synechocystis* sp. PCC 6803
43	Glycn_Met	syn00511	Other glycan degradation - *Synechocystis* sp. PCC 6803
44	Carbo_Met	syn00520	Amino sugar and nucleotide sugar metabolism - *Synechocystis* sp. PCC 6803
45	OSecM_BS	syn00521	Streptomycin biosynthesis - *Synechocystis* sp. PCC 6803
46	TerPk_Met	syn00523	Polyketide sugar unit biosynthesis - *Synechocystis* sp. PCC 6803
47	Glycn_Met	syn00540	Lipopolysaccharide biosynthesis - *Synechocystis* sp. PCC 6803
48	Glycn_Met	syn00550	Peptidoglycan biosynthesis - *Synechocystis* sp. PCC 6803
49	Lipid_Met	syn00561	Glycerolipid metabolism - *Synechocystis* sp. PCC 6803
50	Carbo_Met	syn00562	Inositol phosphate metabolism - *Synechocystis* sp. PCC 6803
51	Lipid_Met	syn00564	Glycerophospholipid metabolism - *Synechocystis* sp. PCC 6803
52	Lipid_Met	syn00590	Arachidonic acid metabolism - *Synechocystis* sp. PCC 6803
53	Carbo_Met	syn00620	Pyruvate metabolism - *Synechocystis* sp. PCC 6803
54	XenBD_Met	syn00623	Toluene degradation - *Synechocystis* sp. PCC 6803
55	XenBD_Met	syn00624	Polycyclic aromatic hydrocarbon degradation - *Synechocystis* sp. PCC 6803
56	XenBD_Met	syn00625	Chloroalkane and chloroalkene degradation - *Synechocystis* sp. PCC 6803

57	XenBD_Met	syn00626	Naphthalene degradation - *Synechocystis* sp. PCC 6803
58	XenBD_Met	syn00627	Aminobenzoate degradation - *Synechocystis* sp. PCC 6803
59	Carbo_Met	syn00630	Glyoxylate and dicarboxylate metabolism - *Synechocystis* sp. PCC 6803
60	Carbo_Met	syn00640	Propanoate metabolism - *Synechocystis* sp. PCC 6803
61	XenBD_Met	syn00643	Styrene degradation - *Synechocystis* sp. PCC 6803
62	Carbo_Met	syn00650	Butanoate metabolism - *Synechocystis* sp. PCC 6803
63	Carbo_Met	syn00660	C5-Branched dibasic acid metabolism - *Synechocystis* sp. PCC 6803
64	CfVit_Met	syn00670	One carbon pool by folate - *Synechocystis* sp. PCC 6803
65	Enrgy_Met	syn00680	Methane metabolism - *Synechocystis* sp. PCC 6803
66	Enrgy_Met	syn00710	Carbon fixation in photosynthetic organisms - *Synechocystis* sp. PCC 6803
67	Enrgy_Met	syn00720*	Carbon fixation pathways in prokaryotes
68	CfVit_Met	syn00730	Thiamine metabolism - *Synechocystis* sp. PCC 6803
69	CfVit_Met	syn00740	Riboflavin metabolism - *Synechocystis* sp. PCC 6803
70	CfVit_Met	syn00750	Vitamin B6 metabolism - *Synechocystis* sp. PCC 6803
71	CfVit_Met	syn00760	Nicotinate and nicotinamide metabolism - *Synechocystis* sp. PCC 6803
72	CfVit_Met	syn00770	Pantothenate and CoA biosynthesis - *Synechocystis* sp. PCC 6803
73	CfVit_Met	syn00780	Biotin metabolism - *Synechocystis* sp. PCC 6803
74	CfVit_Met	syn00785	Lipoic acid metabolism - *Synechocystis* sp. PCC 6803
75	CfVit_Met	syn00790	Folate biosynthesis - *Synechocystis* sp. PCC 6803
76	CfVit_Met	syn00860	Porphyrin and chlorophyll metabolism - *Synechocystis* sp. PCC 6803
77	TerPk_Met	syn00900	Terpenoid backbone biosynthesis - *Synechocystis* sp. PCC 6803
78	TerPk_Met	syn00903	Limonene and pinene degradation - *Synechocystis* sp. PCC 6803

79	TerPk_Met	syn00906	Carotenoid biosynthesis - *Synechocystis* sp. PCC 6803
80	Enrgy_Met	syn00910	Nitrogen metabolism - *Synechocystis* sp. PCC 6803
81	Enrgy_Met	syn00920	Sulfur metabolism - *Synechocystis* sp. PCC 6803
82	Trnsl_GIP	syn00970	Aminoacyl-tRNA biosynthesis - *Synechocystis* sp. PCC 6803
83	MbTrn_Env	syn02010	ABC transporters - *Synechocystis* sp. PCC 6803
84	SigTr_Env	syn02020	Two-component system - *Synechocystis* sp. PCC 6803
85	Trnsl_GIP	syn03010	Ribosome - *Synechocystis* sp. PCC 6803
86	FdDeg_GIP	syn03018	RNA degradation - *Synechocystis* sp. PCC 6803
87	Trnsc_GIP	syn03020	RNA polymerase - *Synechocystis* sp. PCC 6803
88	Replic_GIP	syn03030	DNA replication - *Synechocystis* sp. PCC 6803
89	FdDeg_GIP	syn03060	Protein export - *Synechocystis* sp. PCC 6803
90	MbTrn_Env	syn03070	Bacterial secretion system - *Synechocystis* sp. PCC 6803
91	Replic_GIP	syn03410	Base excision repair - *Synechocystis* sp. PCC 6803
92	Replic_GIP	syn03420	Nucleotide excision repair - *Synechocystis* sp. PCC 6803
93	Replic_GIP	syn03430	Mismatch repair - *Synechocystis* sp. PCC 6803
94	Replic_GIP	syn03440	Homologous recombination - *Synechocystis* sp. PCC 6803
95	FdDeg_GIP	syn04122	Sulfur relay system - *Synechocystis* sp. PCC 6803

*syn00100 and syn00720 are not found in *Synechocystis* sp. PCC 6803 from Kyoto Encyclopedia of Genes and Genomes (KEGG) database.

SpirPro is based on proteomic data and interactome data inference from orthologous proteins in another cyanobacterium, *Synechocystis* sp. PCC 6803, and incorporates this information into KEGG pathways.
