# Supplementary material for: Network analysis retrieving bioactive compounds from Spirulina (Arthrospira platensis) and their targets related to systemic lupus erythematosus
Source: PLoS One. 2024 Aug 29;19(8):e0309303. doi: 10.1371/journal.pone.0309303 (PMC11361558; doi:10.1371/journal.pone.0309303)
Supplement: S2 Table — SpirPro is based on proteomic data and interactome data inference from orthologous proteins in another cyanobacterium, Synechocystis sp. PCC 6803, and incorporates this information into KEGG pathways. (PDF) [file pone.0309303.s003.pdf]

S2 Table. List of 699 enzymes from 95 metabolic pathways of *A. platensis* C1 retrieved from Spirulina-Proteome Repository (SpirPro) database.

No	Entry	EC*				
1	syn00010	5.4.2.2	2.7.1.2	5.3.1.9	3.1.3.11	2.7.1.11
		4.1.2.13	5.3.1.1	1.2.1.12	1.2.1.59	2.7.2.3
		5.4.2.1	4.2.1.11	2.7.1.40	2.3.1.12	1.2.4.1
		6.2.1.1	1.8.1.4	1.2.1.3	1.1.1.1	1.1.1.2
2	syn00020	2.3.1.12	1.2.4.1	1.8.1.4	1.1.1.37	2.3.3.1
		4.2.1.3	1.1.1.42	4.2.1.2	1.3.99.1	6.2.1.5
3	syn00030	1.1.1.47	5.3.1.9	1.1.1.49	3.1.1.31	4.1.2.14
		1.1.1.44	2.2.1.1	5.1.3.1	3.1.3.11	2.7.1.11
		5.3.1.6	2.2.1.2	4.1.2.13	2.7.6.1	5.4.2.2
		4.1.2.4				
4	syn00040	1.1.1.22	4.1.2.14	1.2.1.3	5.1.3.1	
5	syn00051	2.7.1.4	5.3.1.8	5.4.2.8	3.1.3.11	2.7.1.11
		2.7.7.13	2.7.7.22	4.2.1.47	1.1.1.271	4.1.2.13
		5.3.1.1				
6	syn00052	5.1.3.2	5.4.2.2	2.7.1.2	2.7.1.11	
7	syn00053	1.1.1.22	1.2.1.3			
8	syn00061	6.4.1.2	6.3.4.14			
9	syn00071	6.2.1.3	2.3.1.9	1.1.1.1	1.2.1.3	
10	syn00100	2.5.1.21	5.4.99.17			
11	syn00130	1.13.11.27	5.4.4.2	2.2.1.9	4.2.99.20	4.2.1.113
		6.2.1.26	4.1.3.36	3.1.2.28	2.5.1.74	2.5.1.-
		2.1.1.163	2.1.1.-	2.5.1.-	4.1.1.-	
12	syn00190	1.9.3.1	1.3.99.1	3.6.3.14	1.6.5.3	1.6.99.3
		2.7.4.1	3.6.1.1			
13	syn00195	3.6.3.14	1.18.1.2	1.10.99.1		

14	syn00196	-				
15	syn00230	5.4.2.2	3.6.1.13	4.6.1.1	2.7.6.1	2.4.2.14
		3.6.1.11	2.7.7.6	2.7.7.7	6.3.4.13	3.1.5.1
		3.1.7.2	2.7.7.8	2.7.4.6	3.1.3.5	2.1.2.2
		2.7.4.6	1.17.4.1	2.7.4.8	6.3.5.3	3.6.1.19
		2.7.4.8	2.4.2.7	6.3.3.1	6.3.4.18	6.3.5.2
		5.4.99.18	6.3.2.6	1.1.1.205	4.3.2.2	2.1.2.3
		2.4.2.7	6.3.4.4	3.5.1.5	2.7.4.3	1.17.4.1
		2.7.7.53	2.7.7.4	2.7.1.25	3.6.1.15	2.7.4.3
		2.7.2.2				
16	syn00240	2.1.3.2	6.3.5.5	1.8.1.9	3.5.2.3	2.7.7.7
		1.3.98.1	2.7.7.8	2.7.7.6	3.5.4.13	6.3.4.2
		2.7.4.6	2.4.2.10	3.6.1.19	1.17.4.1	4.1.1.23
		2.7.4.14	2.7.4.22	2.7.4.9	2.1.1.148	3.1.3.5
		2.4.2.9	3.5.4.1			
17	syn00250	2.1.3.2	6.3.4.4	6.3.4.5	3.5.1.1	3.5.1.15
		1.4.1.1	4.3.2.2	4.3.2.1	1.4.3.16	2.6.1.1
		1.2.1.16	1.4.1.4	1.4.1.13	1.4.1.14	2.6.1.16
		4.1.1.15	6.3.1.2	3.5.1.2	2.4.2.14	1.5.1.12
		6.3.5.5				
18	syn00260	1.1.1.95	4.3.1.19	4.2.1.20	2.1.2.1	2.1.2.10
		1.8.1.4	1.4.4.2	1.4.3.4	4.3.1.19	4.2.3.1
		2.7.1.39	1.1.1.3	2.7.2.4	1.2.1.11	
19	syn00270	2.3.1.30	2.5.1.47	2.6.1.1	2.7.2.4	1.2.1.11
		1.1.1.3	3.3.1.1	2.1.1.13	2.1.1.37	2.5.1.6
		2.4.2.28	5.3.1.23			
20	syn00280	2.6.1.42	1.8.1.4	2.3.1.9	1.1.1.31	1.2.1.3
21	syn00290	4.3.1.19	2.2.1.6	1.1.1.86	4.2.1.9	2.6.1.42

		6.1.1.6	6.1.1.9	4.2.1.35	1.2.4.1	2.3.3.13
		4.2.1.33	1.1.1.85	2.6.1.42	6.1.1.4	
22	syn00300	1.1.1.3	2.7.2.4	1.2.1.11	4.2.1.52	1.3.1.26
		2.6.1.83	5.1.1.7	6.3.2.10	6.3.2.13	4.1.1.20
		1.5.1.7				
23	syn00310	1.5.1.7	4.1.1.18	2.3.1.9	3.4.-.-	1.2.1.3
24	syn00330	3.5.2.10	6.3.4.5	4.3.2.1	3.5.4.1	3.5.1.2
		6.3.1.2	2.7.2.2	2.1.3.3	4.1.1.19	1.4.1.4
		2.6.1.11	2.3.1.35	3.5.1.5	2.3.1.1	2.7.2.8
		1.2.1.38	1.5.1.12	2.7.2.11	1.2.1.41	1.5.1.12
		1.5.1.2	1.5.99.8	2.6.1.1	4.1.2.14	4.1.3.16
		1.5.1.12	1.5.99.8	3.5.3.11	3.5.1.4	1.4.3.4
		1.2.1.3	4.1.1.-			
25	syn00340	2.4.2.17	3.6.1.3	3.5.4.19	5.3.1.16	4.2.1.19
		2.6.1.9	1.1.1.23	2.1.1.-	3.5.1.15	1.2.1.3
		1.4.3.4				
26	syn00350	1.4.3.4	1.2.1.16	2.1.1.-	2.6.1.1	2.6.1.9
		1.13.11.27	1.4.3.4	1.1.1.1		
27	syn00360	2.6.1.1	2.6.1.9	1.4.3.4	1.11.1.7	1.13.11.27
		3.5.1.4				
28	syn00361	3.1.1.45				
29	syn00362	4.1.1.46	2.3.1.9			
30	syn00363	1.14.-.-				
31	syn00364	3.1.1.45				
32	syn00380	1.2.1.3	1.4.3.4	1.2.1.3	3.5.1.4	1.11.1.6
		2.3.1.9				
33	syn00400	2.5.1.54	4.2.3.4	4.2.1.10	1.1.1.25	4.2.1.20
		2.7.1.71	4.1.1.48	2.5.1.19	5.3.1.24	4.2.3.5

		2.4.2.18	4.1.3.27	5.4.99.5	4.2.1.51	2.6.1.1.
		2.6.1.9	1.3.1.78			
34	syn00401	2.6.1.1	2.6.1.9			
35	syn00410	4.1.1.11	4.1.1.15	1.2.1.3	6.3.2.1	
36	syn00430	4.1.1.15	2.3.2.2	1.4.1.1	2.3.1.8	2.7.2.1
37	syn00450	6.1.1.10	1.8.1.9	4.4.1.16	1.8.1.9	2.7.7.4
38	syn00460	3.5.1.4	3.2.1.21	2.1.2.1	2.3.2.2	3.5.1.1
39	syn00471	3.5.1.2	5.1.1.3	6.3.2.9	6.3.2.8	
40	syn00473	5.1.1.1	6.3.2.4			
41	syn00480	3.5.2.9	3.4.11.2	2.3.2.2	6.3.2.3	1.1.1.42
		1.1.1.44	1.1.1.49	2.5.1.18	1.11.1.9	
42	syn00500	2.4.1.14	1.1.1.22	3.2.1.21	2.7.7.27	2.4.1.21
		2.7.1.4	3.2.1.4	2.4.1.1	2.4.1.25	2.7.7.33
		2.4.1.18	5.4.2.2	2.7.1.2	5.3.1.9	
43	syn00511	-				
44	syn00520	5.1.3.8	4.2.-.-	2.7.7.23	5.1.3.14	2.3.1.157
		5.4.2.10	5.1.3.14	2.6.1.16	2.7.1.4	2.5.1.7
		1.1.1.158	5.3.1.9	1.1.1.22	5.4.2.2	2.7.1.2
		5.3.1.8	5.1.3.2	3.13.1.1	5.4.2.8	2.7.7.13
		2.7.7.22	4.2.1.47	1.1.1.271	2.7.1.2	2.7.7.27
45	syn00521	2.7.1.2	5.4.2.2	3.1.3.25	2.7.7.24	4.2.1.46
		5.1.3.13	1.1.1.133			
46	syn00523	2.7.7.24	4.2.1.46	5.1.3.13	1.1.1.133	
47	syn00540	2.3.1.129	2.4.1.182			
48	syn00550	2.5.1.7	1.1.1.158	6.3.2.8	6.3.2.9	6.3.2.10
		2.7.8.13	2.4.1.227	6.3.2.4	3.6.1.27	6.3.2.13
		3.4.16.4				
49	syn00561	1.2.1.3	1.1.1.2	1.1.1.6	2.7.1.30	2.3.1.15

		2.3.1.51	2.7.1.107	3.1.1.3	2.4.1.157	
50	syn00562	3.1.3.25	5.3.1.1			
51	syn00564	1.1.1.94	1.1.5.3	2.3.1.15	2.7.1.107	2.7.7.41
		2.7.8.5				
52	syn00590	2.3.2.2	1.11.1.9			
53	syn00620	4.2.3.3	4.1.1.31	2.7.9.2	4.4.1.5	3.1.2.6
		2.7.1.40	1.1.1.28	1.2.4.1	1.1.1.38	2.7.2.1
		2.3.1.8	1.8.1.4	2.3.1.12	1.1.1.37	1.2.1.3
		6.2.1.1	2.3.3.13	2.3.1.9	6.4.1.2	
54	syn00623	1.3.99.1	3.1.1.45			
55	syn00624	1.14.-.-	2.1.1.-			
56	syn00625	1.1.1.1	1.2.1.3			
57	syn00626	1.1.1.1				
58	syn00627	1.14.-.-	3.5.1.4	4.1.1.46		
59	syn00630	2.3.3.1	4.2.1.3	2.3.1.9	1.1.1.37	3.5.1.10
		4.1.1.2	1.1.3.15	4.1.3.16	4.1.1.39	3.1.3.18
60	syn00640	2.7.2.1	2.3.1.8	6.2.1.1	6.4.1.2	2.3.1.9
		1.2.1.3	6.2.1.5			
61	syn00643	3.5.1.4	3.5.5.7			
62	syn00650	2.2.1.6	1.2.4.1	1.3.99.1	1.2.1.16	4.1.1.15
		2.3.1.-	2.3.1.9			
63	syn00660	6.2.1.5	2.2.1.6	4.2.1.35		
64	syn00670	2.1.1.148	2.1.2.10	2.1.2.1	2.1.2.3	2.1.2.2
		3.5.1.10	2.1.2.9	2.1.1.13	3.5.4.9	6.3.3.2
		1.5.1.5				
65	syn00680	6.2.1.1	2.7.9.2	4.2.1.11	5.4.2.1	1.1.1.95
		4.1.1.31	1.1.1.37	4.1.2.13	3.1.3.11	2.7.1.11
		2.1.2.1	1.11.1.6	1.11.1.7	3.1.2.12	1.1.1.284

		2.5.1.77	1.12.98.1	2.7.2.1	2.3.1.8	6.2.1.1
		3.1.3.71	1.8.98.1			
66	syn00710	4.1.2.13	2.2.1.1	3.1.3.37	4.1.2.13	5.3.1.1
		2.2.1.1	1.2.1.59	5.3.1.6	5.1.3.1	2.7.1.19
		2.7.2.3	4.1.1.39	4.1.1.31	2.7.1.40	2.6.1.1
		1.1.1.37				
67	syn00720	-				
68	syn00730	2.8.1.7	2.5.1.3	2.7.4.16	3.6.1.15	
69	syn00740	4.1.99.12	3.5.4.25	3.5.4.26	1.1.1.193	2.5.1.78
		2.5.1.9	2.7.1.26	2.7.7.2		
70	syn00750	1.4.3.5	2.6.99.2	1.1.1.262	4.2.3.1	
71	syn00760	2.4.2.12	3.1.3.5	2.7.7.1	2.7.7.18	1.4.3.16
		6.3.1.5	2.7.1.23	1.6.1.2		
72	syn00770	2.2.1.6	1.1.1.86	4.2.1.9	2.1.2.11	2.6.1.42
		6.3.2.1	2.7.1.33	6.3.2.5	4.1.1.36	4.1.1.11
		2.7.7.3	2.7.8.-	2.7.1.24		
73	syn00780	2.3.1.47	6.3.3.3	2.8.1.6	6.3.4.15	3.4.-.-
74	syn00785	2.8.1.8	2.3.1.181	2.7.7.63		
75	syn00790	3.5.4.16	4.2.3.12	4.1.2.25	2.7.6.3	4.1.3.38
		2.5.1.15	6.3.2.17	6.3.2.12		
76	syn00860	4.1.1.37	4.2.1.24	2.5.1.61	4.2.1.75	1.3.3.3
		1.3.99.22	4.99.1.1	5.4.3.8	1.2.1.70	6.1.1.17
		4.99.1.3	2.1.1.107	2.5.1.17	6.3.5.10	2.7.1.156
		6.3.1.10	2.7.7.62	2.7.8.26	2.5.1.17	2.5.1.-
		6.6.1.1	2.1.1.11	1.14.13.81	1.3.1.33	1.18.-.-
		2.5.1.62	1.14.99.3	1.3.7.5	1.3.1.24	
77	syn00900	2.3.1.9	2.2.1.7	1.1.1.267	2.7.7.60	2.7.1.148
		4.6.1.12	1.17.7.1	1.17.1.2	5.3.3.2	2.5.1.1

		2.5.1.31	2.5.1.10	2.5.1.29	1.3.1.83	2.5.1.85
		2.5.1.84				
78	syn00903	1.14.-.-	1.2.1.3			
79	syn00906	2.5.1.32	1.14.99.-	1.14.99.30		
80	syn00910	4.2.1.1	4.2.1.104	2.7.2.2	6.3.1.5	2.1.2.10
		3.5.1.1	1.7.7.2	1.7.7.1	6.3.1.2	3.5.1.2
		1.4.1.13	1.4.1.14	1.4.7.1	1.4.1.4	
81	syn00920	2.3.1.30	2.5.1.47	2.7.7.4	1.8.7.1	2.7.1.25
		1.8.4.8				
82	syn00970	6.1.1.17	6.3.5.7	6.1.1.7	6.1.1.12	6.3.5.6
		6.1.1.22	6.1.1.14	6.1.1.3	6.1.1.11	6.1.1.16
		6.1.1.10	2.1.2.9	6.1.1.9	6.1.1.4	6.1.1.5
		6.1.1.6	6.1.1.19	6.1.1.15	6.1.1.21	6.1.1.20
		6.1.1.1	6.1.1.2			
83	syn02010	-				
84	syn02020	-				
85	syn03010	-				
86	syn03018	-				
87	syn03020	-				
88	syn03030	-				
89	syn03060	-				
90	syn03070	-				
91	syn03410	-				
92	syn03420	-				
93	syn03430	-				
94	syn03440	-				
95	syn04122	-				

*EC; enzyme number in Kyoto Encyclopedia of Genes and Genomes (KEGG) database

SpirPro is based on proteomic data and interactome data inference from orthologous proteins in another cyanobacterium, *Synechocystis* sp. PCC 6803, and incorporates this information into KEGG pathways.
