# Supplementary material for: Network analysis retrieving bioactive compounds from Spirulina (Arthrospira platensis) and their targets related to systemic lupus erythematosus
Source: PLoS One. 2024 Aug 29;19(8):e0309303. doi: 10.1371/journal.pone.0309303 (PMC11361558; doi:10.1371/journal.pone.0309303)
Supplement: S3 Table — (PDF) [file pone.0309303.s004.pdf]

S3 Table. List of 833 compounds retrieved from *A. platensis* C1, along with their corresponding compound identifier numbers in the PubChem database.

No	CID*	Compound Name
1	5	3-AMINO-2-OXOPROPYL PHOSPHATE
2	22	2-ACETOLACTATE
3	38	2-DEHYDROPANTOATE
4	46	AC1Q6RYN
5	49	3-METHYL-2-OXOBUTANOIC ACID
6	51	2-KETOGLUTARIC ACID
7	58	2-OXOBUTANOIC ACID
8	59	GLYCERATE 2-PHOSPHATE
9	70	4-METHYL-2-OXOPENTANOIC ACID
10	77	2-ISOPROPYLMALIC ACID
11	105	PHOSPHOHYDROXYPYRUVIC ACID
12	118	4-AMINOBUTANAL
13	119	4-AMINOBUTYRIC ACID
14	125	4-HYDROXYBENZYL ALCOHOL
15	127	4-HYDROXYPHENYLACETIC ACID
16	135	4-HYDROXYBENZOIC ACID
17	137	5-AMINOLEVULINIC ACID
18	170	DIHYDROPTEROATE
19	173	8-AMINO-7-OXONONANOIC ACID
20	175	ACETATE
21	176	ACETIC ACID
22	177	ACETALDEHYDE
23	186	ACETYLPHOSPHATE
24	190	ADENINE
25	192	ADPRIBOSE
26	195	ADENYLOSUCCINIC ACID
27	199	AGMATINE
28	200	AICA-RIBOSIDE, 5'-PHOSPHATE
29	216	(4-AMINO-2-METHYLPYRIMIDIN-5-YL)METHYL DIHYDROGEN PHOSPHATE
30	217	(4-AMINO-2-METHYLPYRIMIDIN-5-YL)METHYL TRIHYDROGEN DIPHOSPHATE
31	218	6-HYDROXYMETHYL-7,8-DIHYDROPTERIN

32	222	AMMONIA
33	223	AMMONIUM
34	227	ANTHRANILIC ACID
35	234	ARSENIC ACID
36	239	BETA-ALANINE
37	271	CALCIUM ION
38	273	PENTANE-1,5-DIAMINE
39	277	CARBAMIC ACID
40	278	CARBAMOYLPHOSPHATE
41	280	CARBON DIOXIDE
42	281	CARBON MONOXIDE
43	284	FORMIC ACID
44	289	PYROCATECHOL
45	296	METHYLMALONATE SEMIALDEHYDE
46	297	METHANE
47	311	CITRIC ACID
48	321	COPROPORPHYRINOGEN III
49	329	(3,4-DIHYDROXY-5-METHYLSULFANYL-2-OXO-PENTOXY)KPHOSPHONIC ACID
50	389	2,5-DIAMINOPENTANOIC ACID
51	402	HYDROGEN SULFIDE
52	424	DL-ASPARTIC ACID
53	441	3-HYDROXYBUTYRIC ACID
54	451	GDP-FUC
55	473	4-METHYLTHIO-2-OXOBUTANOIC ACID
56	525	MALIC ACID
57	529	PHOSPHOGLYCOLIC ACID
58	533	HYDROGEN SELENIDE
59	540	CYANIC ACID
60	544	ARSENITE ION
61	547	3,4-DIHYDROXYPHENYLACETIC ACID
62	553	4-HYDROXY-2-OXOHEPTANEDIOIC ACID
63	561	5-(METHYLTHIO)-2,3-DIOXOPENTYL PHOSPHATE
64	563	3-(METHYLTHIO)PROPIONIC ACID
65	586	CREATINE
66	588	CREATININE
67	595	DL-CYSTINE

68	597	CYTOSINE
69	598	2-MERCAPTOETHANESULFONIC ACID
70	611	DL-GLUTAMIC ACID
71	632	THDPA
72	636	DESOXYADENOSINE
73	638	DESOXYGUANOSINE
74	643	DESTHIOBIOTIN
75	647	DMAPP
76	652	7,8-DIAMINONONANOIC ACID
77	666	PTERIDINE DIPHOSPHATE
78	668	DIHYDROXYACETONE PHOSPHATE
79	669	2-HYDROXY-3-OXOBUTYL PHOSPHATE
80	670	1,3-DIHYDROXYACETONE
81	671	1,4-DIHYDROXY-2-NAPHTHOIC ACID
82	675	5,6-DIMETHYLBENZIMIDAZOLE
83	683	1,3-BIPHOSPHOGLYCERATE
84	702	ETHANOL
85	713	FORMAMIDE
86	729	GLYCERALDEHYDE 3-PHOSPHATE
87	750	GLYCINE
88	753	GLYCEROL
89	754	GLYCEROPHOSPHORIC ACID
90	756	GLYCOLALDEHYDE
91	757	GLYCOLIC ACID
92	760	GLYOXYLIC ACID
93	764	GUANINE
94	767	CARBONIC ACID
95	769	BICARBONATE
96	779	DL-HOMOSERINE
97	780	HOMOGENTISIC ACID
98	783	HYDROGEN
99	784	HYDROGEN PEROXIDE
100	785	HYDROQUINONE
101	788	HYDROXYMETHYLBILANE
102	792	IMIDAZOLE ACETOL PHOSPHATE
103	796	IMINOASPARTATE

104	798	INDOLE
105	807	IODINE
106	813	POTASSIUM ION
107	850	5-O-PHOSPHONOPENT-2-ULOSE
108	864	DL-THIOCTIC ACID
109	871	MALTOTRIOSE
110	880	METHYLGLYOXAL
111	888	MAGNESIUM ION
112	892	MYO-INOSITOL
113	905	2-ACETAMIDO-5-OXOVALERIC ACID
114	923	SODIUM ION
115	935	NICKEL
116	936	NICOTINAMIDE
117	938	NICOTINIC ACID
118	940	3-CARBAMOYL-1-PENTOFURANOSYLPYRIDINIUM
119	944	NITRIC ACID
120	955	2-SUCCINYLBENZOATE
121	956	2-SUCCINYLBENZOYL-COENZYME A
122	962	WATER
123	964	3-HYDROXYPYRUVIC ACID
124	967	OROTIC ACID
125	970	OXALACETIC ACID
126	971	OXALIC ACID
127	972	OXALOSUCCINIC ACID
128	977	OXYGEN
129	978	4-AMINOBENZOIC ACID
130	979	4-HYDROXYPHENYLPYRUVIC ACID
131	983	TRIPHOSPHORIC ACID
132	985	PALMITIC ACID
133	986	AC1L1AH5
134	994	DL-PHENYLALANINE
135	996	PHENOL
136	997	PHENYLPYRUVIC ACID
137	1004	PHOSPHORIC ACID
138	1005	PHOSPHOENOLPYRUVATE
139	1021	PORPHOBILINOGEN

140	1023	DIPHOSPHORIC ACID
141	1028	PREPHENIC ACID
142	1032	PROPIONIC ACID
143	1034	PROPANOYL PHOSPHATE
144	1038	HYDRON
145	1045	1,4-DIAMINOBUTANE
146	1051	PYRIDOXAL PHOSPHATE
147	1055	PYRIDOXINE PHOSPHATE
148	1060	PYRUVIC ACID
149	1066	QUINOLINIC ACID
150	1071	9-CIS RETINOL
151	1090	SELENITE
152	1100	SULFUROUS ACID
153	1102	SPERMIDINE
154	1103	SPERMINE
155	1110	SUCCINIC ACID
156	1112	SUCCINIC SEMIALDEHYDE
157	1117	SULFATE
158	1118	SULFURIC ACID
159	1130	THIAMINE
160	1131	THIAMIN PHOSPHATE
161	1132	COCARBOXYLASE
162	1135	THYMINE
163	1137	4-METHYL-5-HYDROXYETHYLTHIAZOLE PHOSPHATE
164	1174	URACIL
165	1176	UREA
166	1179	UROPORPHYRINOGEN III
167	1188	XANTHINE
168	1195	ISOPENTENYL PYROPHOSPHATE
169	1198	ISOCITRIC ACID
170	3073	DIISOPROPYL METHYLPHOSPHONATE
171	4971	PROTOPORPHYRIN IX
172	5281	STEARIC ACID
173	5779	D-RIBOFURANOSE
174	5789	THYMIDINE
175	5793	D-GLUCOSE

176	5862	L-CYSTEINE
177	5884	NADPH
178	5886	NADP+
179	5893	NICOTINAMIDE ADENINE DINUCLEOTIDE
180	5950	L-ALANINE
181	5951	L-SERINE
182	5957	ADENOSINE TRIPHOSPHATE
183	5958	ROBISON ESTER
184	5960	L-ASPARTIC ACID
185	5961	L-GLUTAMINE
186	5962	L-LYSINE
187	5984	D-(-)-FRUCTOSE
188	5988	SUCROSE
189	6019	PYRIDOXINE HYDROCHLORIDE
190	6021	INOSINE
191	6022	ADENOSINE 5'-DIPHOSPHATE
192	6029	URIDINE
193	6030	URIDINE 5'-MONOPHOSPHATE
194	6031	URIDINE 5'-DIPHOSPHATE
195	6036	D-GALACTOSE
196	6037	FOLIC ACID
197	6057	L-TYROSINE
198	6076	CAMP
199	6083	ADENOSINE 5'-MONOPHOSPHATE
200	6106	L-LEUCINE
201	6131	5'-CYTIDYLIC ACID
202	6132	CYTIDINE 5'-DIPHOSPHATE
203	6133	URIDINE 5'-TRIPHOSPHATE
204	6137	L-METHIONINE
205	6140	L-PHENYLALANINE
206	6175	CYTIDINE
207	6176	CYTIDINE TRIPHOSPHATE
208	6202	THIAMINE HYDROCHLORIDE
209	6262	L-ORNITHINE
210	6267	L-ASPARAGINE
211	6274	L-HISTIDINE

212	6287	L-VALINE
213	6288	L-THREONINE
214	6305	L-TRYPTOPHAN
215	6306	L-ISOLEUCINE
216	6322	L-ARGININE
217	6613	D-PANTOTHENIC ACID
218	6802	GUANOSINE
219	6804	5'-GUANYLIC ACID
220	6830	GUANOSINE TRIPHOSPHATE
221	6831	INOSINE DIPHOSPHATE
222	7027	GLUCONOLACTONE
223	7339	PRPP
224	7405	L-PYROGLUTAMIC ACID
225	7427	TREHALOSE
226	8299	HYDROXYACETONE
227	8582	INOSINIC ACID
228	8583	INOSINE TRIPHOSPHATE
229	8629	UDP-GLUCOSE
230	8742	SHIKIMIC ACID
231	8892	HEXANOIC ACID
232	8965	VITAMIN B2 PHOSPHATE
233	8977	GUANOSINE 5'-DIPHOSPHATE
234	8989	(2R)-2,5,8-TRIMETHYL-2-(4,8,12-TRIMETHYLTRIDECYL)CHROMAN-6-OL
235	9700	THYMIDYLIC ACID
236	9750	L-CITRULLINE
237	10214	PAPS
238	10238	ADENYLYL SULFATE
239	10267	BETA-D-FRUCTOSE 1,6-BISPHOSPHATE
240	10465	HEPTADECANOIC ACID
241	10663	OMEGA-CARBOXYACYL-COA
242	11005	TETRADECANOIC ACID
243	12039	CHORISMIC ACID
244	12599	DAMP
245	12647	L-HOMOSERINE
246	13945	DEOXYCYTIDINE 5'-MONOPHOSPHATE
247	14180	BETA-NICOTINAMIDE MONONUCLEOTIDE

248	14985	VITAMIN E
249	14986	TOCOPHEROLS
250	15993	DATP
251	16500	ADP-GLUCOSE
252	16950	ARGININOSUCCINIC ACID
253	17473	UDP-GLUCURONIC ACID
254	18068	UDP GALACTOSE
255	18396	GDP-D-MANNOSE
256	19233	RHAMNOSE
257	20353	VITAMIN E SUCCINATE
258	21706	DIADENOSINE TETRAPHOSPHATE
259	23327	D-GLUTAMIC ACID
260	23925	IRON
261	23930	MANGANESE
262	23932	MOLYBDENUM
263	23973	CADMIUM
264	23976	CHROMIUM
265	23978	COPPER
266	23994	ZINC
267	24316	CYCLIC GMP
268	24404	PHOSPHINE
269	24529	NITROUS ACID
270	26623	MERCURIC ION
271	26945	HEME
272	27284	FERROUS ION
273	29936	FERRIC ION
274	33032	L-GLUTAMIC ACID
275	34755	ADEMETIONINE
276	38166	GUANOSINE PENTAPHOSPHATE
277	41635	PHOSPHOPANTOTHENIC ACID
278	60961	ADENOSINE
279	61503	D-LACTIC ACID
280	62698	DEXTRIN
281	64689	BETA-D-GLUCOSE
282	64959	XANTHOSINE
283	64968	DTTP

284	65040	5-METHYLCYTOSINE
285	65058	2'-DEOXYINOSINE
286	65059	DEOXYGUANYLIC ACID
287	65063	DUMP
288	65065	N-ACETYL-L-ASPARTIC ACID
289	65070	DUTP
290	65074	7,8-DIHYDRONEOPTERIN
291	65091	DCTP
292	65103	DGTP
293	65110	AICA RIBONUCLEOTIDE
294	65127	MANNOSE 6-PHOSPHATE
295	65246	FRUCTOSE-1-PHOSPHATE
296	65270	GLYCINE, N-CYSTEINYL
297	65359	OXIGLUTATIONE
298	65533	GLUCOSE-1-PHOSPHATE
299	68499	NICOTINURIC ACID
300	68589	ECONAZOLE NITRATE
301	68841	O-PHOSPHO-L-SERINE
302	70914	N-ACETYL-L-GLUTAMIC ACID
303	71080	D-ALANINE
304	72423	UROPORPHYRIN III
305	73323	XANTHOSINE MONOPHOSPHATE
306	77982	RIBOSE-5-PHOSPHATE
307	79025	ALPHA-D-GLUCOSE
308	82208	MOLYBDIC ACID
309	87642	COENZYME A
310	91443	TETRAHYDROFOLIC ACID
311	91493	6-PHOSPHOGLUCONIC ACID
312	91552	L-HOMOCYSTEINE
313	92094	DELTA-TOCOPHEROL
314	92133	SUCCINYL-COENZYME A
315	92153	ACETOACETYL-COA
316	92155	DIPLOPTENE
317	92729	GAMMA-TOCOPHEROL
318	92823	2',3'-CYCLIC GMP
319	92824	D-(+)-MALIC ACID

320	93072	N-CARBAMOYL-L-ASPARTATE
321	98792	DIHYDROFOLIC ACID
322	99290	(2R,6S)-2,6-DIAMINOHEPTANEDIOIC ACID
323	99478	O-ACETYL-L-SERINE
324	104729	COBALT ION
325	107674	SELENIDE
326	107689	L-LACTIC ACID
327	114935	COPROPORPHYRIN III
328	115254	4'-PHOSPHOPANTETHEINE
329	119055	7,8-DIHYDROBIOPTERIN
330	121885	DIHYDRONEOPTERIN TRIPHOSPHATE
331	121893	PROTOPORPHYRINOGEN
332	121947	SHIKIMATE-3-PHOSPHATE
333	121966	DTDP-L-RHAMNOSE
334	121991	NICOTINATE MONONUCLEOTIDE
335	121992	NICOTINATE MONONUCLEOTIDE
336	122280	AC1L3UAV
337	122336	TREHALOSE 6-PHOSPHATE
338	122347	10-FTHF
339	122357	D-ERYTHROSE 4-PHOSPHATE
340	123658	1,5-DI-O-PHOSPHONO-D-RIBULOSE
341	123732	D-RIBOSE 1-PHOSPHATE
342	123907	LIPID X
343	123912	GALACTOSE-1-PHOSPHATE
344	123938	GAMMA-GLUTAMYLCYSTEINE
345	124886	GLUTATHIONE
346	125437	AC1L2OKP
347	126747	2C-METHYL-D-ERYTHRITOL 2,4-CYCLODIPHOSPHATE
348	128973	DYSPROPTERIN
349	129297	GLUTAMATE-1-SEMIALDEHYDE
350	130805	5'-PHOSPHORIBOSYL-N-FORMYLGLYCINAMIDE
351	145729	DUDP
352	145742	L-PROLINE
353	146302	DITP
354	149436	LEVOLEUCOVORIN
355	150855	DCDP

356	151187	O-PHOSPHOHOMOSERINE
357	152441	ASPARTYL PHOSPHATE
358	152657	HISTIDINAL
359	159296	ADENOSINE 3',5'-BISPHOSPHATE
360	159448	PHOSPHINO, SELENOXO-
361	160433	ALPHA-RIBAZOLE
362	160617	OROTIDYLIC ACID
363	160647	DAHP
364	160666	SAICAR
365	160913	GLYCINEAMIDE RIBONUCLEOTIDE
366	161233	NICOTINIC ACID RIBOSIDE
367	161500	AMINOIMIDAZOLE RIBOTIDE
368	164628	DEOXY-TDP
369	164735	SEDOHEPTULOSE 1,7-BISPHOSPHATE
370	165007	SEDOHEPTULOSE 7-PHOSPHATE
371	165271	L-HISTIDINOL
372	165388	CARBOXYAMINOIMIDAZOLE RIBOTIDE
373	165491	DEAMIDO-NAD+
374	166760	FAICAR
375	168989	6,7-DIMETHYL-8-RIBITYLLUMAZINE
376	171548	BIOTIN
377	172313	D-FRUCTOSE-1,6-DIPHOSPHATE
378	172502	UDPGNAC-ENOLPYRUVATE
379	174549	S-METHYL-5-THIO-D-RIBULOSE 1-PHOSPHATE
380	187790	2'-DEOXYGUANOSINE
381	188960	ALPHA-D-GLUCOSAMINE 1-PHOSPHATE
382	188966	DADP
383	189062	ISOCHORISMIC ACID
384	189122	S-FORMYLGLUTATHIONE
385	192878	N-ACETYL-L-GLUTAMATE 5-SEMIALDEHYDE
386	193305	L-GLUTAMATE 5-SEMIALDEHYDE
387	193475	GAMMA-GLUTAMYL PHOSPHATE
388	193516	5-AMINO-6-(D-RIBITYLAMINO)URACIL
389	193533	DIVINYL PROTOCHLOROPHYLLIDE
390	193735	PRFAR
391	194791	UDP-3-HMAGLC

392	196427	SIROHYDROCHLORIN
393	222656	L-MALIC ACID
394	439153	NADH
395	439155	S-ADENOSYLHOMOCYSTEINE
396	439156	URIDINEDIPHOSPHOGLUCOSE
397	439160	NEUBERG ESTER
398	439162	SN-GLYCEROL 3-PHOSPHATE
399	439163	D-FRUCTOFURANOSE
400	439164	N-PROPIONYL COENZYME A LITHIUM SALT
401	439167	D-RIBOSE-5-P
402	439168	GLYCERALDEHYDE-3-PHOSPHATE
403	439175	5,10-METHYLENE-THF
404	439176	5'-METHYLTHIOADENOSINE
405	439177	GLYCOGEN
406	439178	CELLOBIOSE
407	439182	5'-DEOXYADENOSINE
408	439183	3-PHOSPHO-D-GLYCERATE
409	439184	RIBULOSE 5-PHOSPHATE
410	439186	MALTOSE
411	439190	D-XYLULOSE 5-PHOSPHATE
412	439191	1,3-BIPHOSPHOGLYCERIC ACID
413	439194	D-GLYCERIC ACID
414	439197	N-ACETYLNEURAMINIC ACID
415	439208	AC1L96VZ
416	439216	L-DIHYDROOROTIC ACID
417	439217	GLUCOSAMINE 6-PHOSPHATE
418	439220	DGDP
419	439232	N-ACETYLORNITHINE
420	439235	(S)-2-AMINO-4-OXOBUTANOIC ACID
421	439236	RIBOSE 1-PHOSPHATE
422	439237	5,10-METHENYL-TETRAHYDROFOLATE
423	439244	CDP-GLUCOSE
424	439251	PANTOIC ACID
425	439258	L-CYSTATHIONINE
426	439273	3-DEHYDRO-L-GULONATE
427	439278	2-PHOSPHO-D-GLYCERIC ACID

428	439279	ALPHA-D-MANNOSE 1-PHOSPHATE
429	439283	(2S,6S)-2,6-DIAMINOHEPTANEDIOIC ACID
430	439286	ALPHA-KETO-ISOLEUCINE
431	439288	2-DEOXY-D-RIBOFURANOSE 5-PHOSPHATE
432	439292	DTDP-4-DEHYDRO-6-DEOXY-ALPHA-D-GLUCOSE
433	439303	SIROHEME
434	439351	3-DEHYDROQUINIC ACID
435	439398	L-HISTIDINOL PHOSPHATE
436	439399	L-RIBULOSE 5-PHOSPHATE
437	439406	O-SUCCINYL-L-HOMOSERINE
438	439415	S-ADENOSYLMETHIONINAMINE
439	439443	CDP-4-DEHYDRO-6-DEOXY-D-GLUCOSE
440	439446	GDP-4-KETO-6-DEOXYMANNOSE
441	439450	GUANOSINE-5',3'-TETRAPHOSPHATE
442	439452	6-PHOSPHOGLUCONOLACTONE
443	439462	5-AMINO-6-(5-PHOSPHORIBOSYLAMINO)URACIL
444	439463	EPSP
445	439488	DIDP
446	439498	CYSTEINYLGLYCINE
447	439606	MALTOHEXAOSE
448	439664	CHLOROPHYLLIDE A
449	439709	BETA-D-FRUCTOSE
450	439762	SUCROSE 6F-PHOSPHATE
451	439774	3-DEHYDROSHIKIMATE
452	439833	PROTOCHLOROPHYLLIDE
453	439846	(S)-(+)-1,2-PROPANEDIOL
454	439905	5-PHOSPHORIBOSYLAMINE
455	439965	INOSINE-5'-CARBOXYLATE
456	439982	DIHYDRODIPICOLINIC ACID
457	440018	S-D-LACTOYLGLUTATHIONE
458	440035	MAGNESIUM PROTOPORPHYRIN
459	440162	(S)-1-PYRROLINE-5-CARBOXYLATE
460	440179	L-2,3,4,5-TETRAHYDRODIPICOLINIC ACID
461	440194	INOSITOL 3-PHOSPHATE
462	440233	(R)-1-AMINOPROPAN-2-YL PHOSPHATE
463	440236	N-ACETYL-L-GLUTAMYL 5-PHOSPHATE

464	440272	N-ACETYL-D-GLUCOSAMINE 1-PHOSPHATE
465	440279	(R)-2,3-DIHYDROXY-ISOVALERATE
466	440289	NPRAT
467	440304	PANTOTHENYLCYSTEINE 4'-PHOSPHATE
468	440364	ALPHA-GLCNAC-1-P
469	440377	MG-PROTOPORPHYRIN IX 13-MONOMETHYL ESTER
470	440431	D-ERYTHRO-IMIDAZOLE-GLYCEROL PHOSPHATE
471	440568	2-HYDROXYETHYL-THPP
472	440610	KETOHEXANOYL-COA
473	440641	BETA-D-FRUCTOSE 6-PHOSPHATE
474	440721	AC1L99TV
475	440758	ADENYLYLSELENATE
476	440772	GLUTATHIONYLSPERMIDINE
477	440840	FORMAMIDOPYRIMIDINE NUCLEOSIDE TRIPHOSPHATE
478	440841	2,5-DIAMINOPYRIMIDINE NUCLEOSIDE TRIPHOSPHATE
479	440867	ACETYL ADENYLATE
480	440875	(S)-2-ACETO-2-HYDROXYBUTANOATE
481	440878	(S)-2-ACETOLACTATE
482	440901	4-(PHOSPHONOOXY)-THREONINE
483	440923	2,5-DIAMINO-6-(5'-TRIPHOSPHORYL-3',4'-TRIHYDROXY-2'-OXOPENTYL)-AMINO-4-OXOPYRIMIDINE
484	440995	LACTOSE
485	440996	N-ACETYL-D-GLUCOSAMINE 6-PHOSPHATE
486	440997	D-GLUCOSAMINE 6-PHOSPHATE
487	441260	3-OXO-O-PHOSPHONO-L-HOMOSERINE
488	441696	CITRAMALIC ACID
489	442163	TETRAHYDROFOLYL-[GLU](2)
490	443142	4-AMINO-4-DEOXYCHORISMIC ACID
491	443198	METHYL-D-ERYTHRITOL PHOSPHATE
492	443199	CHEBI:16578
493	443200	4-DIPHOSPHOCYTIDYL-2-C-METHYL-D-ERYTHRITOL 2-PHOSPHATE
494	443201	1-DEOXY-D-XYLULOSE 5-PHOSPHATE
495	443210	DTDP-GLUCOSE
496	443211	DTDP-4-KETO-L-RHAMNOSE
497	443244	UDP-6-SULFOQUINOVOSE
498	443249	(2R)-PHOSPHOSULFOLACTATE

499	443250	(2R)-3-SULFOLACTATE
500	443425	C11829
501	443427	C11830
502	443429	2,4-DIVINYLPROTOCHLOROPHYLLIDE
503	443431	DIVINYL CHLOROPHYLLIDE A
504	443753	CALCIUM PANTOTHENATE
505	444097	HEME
506	444150	INDOLE-3-GLYCEROL PHOSPHATE
507	444210	THIOPALMITIC ACID
508	444331	MOLYBDOPTERIN
509	444412	LEVOMEFOLIC ACID
510	444485	DEPHOSPHO-COA
511	444493	ACETYL-COA
512	444848	AC1L9GZX
513	444899	ARACHIDONIC ACID
514	444941	ALPHA-RIBAZOLE-5'-P
515	444972	FUMARIC ACID
516	445117	(6R)-5,10-METHYLENETETRAHYDROFOLATE
517	445557	FRUCTOSE 1,6-BISPHOSPHATE
518	445638	PALMITOLEIC ACID
519	445639	OLEIC ACID
520	445675	UDP-N-ACETYLGLUCOSAMINE
521	445713	FARNESYL DIPHOSPHATE
522	445995	GERANYL DIPHOSPHATE
523	446013	FADH2
524	446872	CEPHALIN
525	446894	1-(2-CARBOXYPHENYLAMINO)-1-DEOXY-D-RIBULOSE 5-PHOSPHATE
526	446925	LYCOPENE
527	447145	ASPARTYL ADENYLATE
528	447277	GERANYLGERANYL PYROPHOSPHATE
529	447633	OCTANETHIOIC ACID
530	448154	2,3-DIHYDROXY-VALERIANIC ACID
531	448265	FE2/S2 (INORGANIC) CLUSTER
532	448348	REDUCED COENZYME F420
533	448701	7,8-DIDEMETHYL-8-HYDROXY-5-DEAZARIBOFLAVIN
534	449043	UDP-MANNAC

535	449118	HEXANOYL-COA
536	449304	4-PHOSPHO-D-ERYTHRONATE
537	449538	URIDINE-5'-DIPHOSPHATE-N-ACETYLMURAMOYL-L-ALANINE-D-GLUTAMATE
538	493570	RIBOFLAVIN
539	638072	SQUALENE
540	643757	CIS-ACONITIC ACID
541	643798	CITRACONIC ACID
542	643975	FLAVIN ADENINE DINUCLEOTIDE
543	643976	FLAVIN MONONUCLEOTIDE
544	644066	MALONYL-COA
545	644175	D-MANNOSE 1-PHOSPHATE
546	656503	UDP-N-ACETYL-BETA-D-MANNOSAMINOURONATE
547	1549100	L,L-DIAMINOPICOLINATE
548	3018065	1,2-DIACYL-SN-GLYCEROL 3-PHOSPHATE
549	3035036	PEPTIDOGLYCAN PENTAPEPTIDE-MDAP3
550	3080745	KDPG INTERMEDIATE
551	3082140	PIMELOYL-COA
552	3423467	CARBAMOYL PHOSPHATE
553	3541112	3-HYDROXYBUTYRATE
554	3593277	2-KETOBUTYRATE
555	3674425	PHOSPHOENOL PYRUVATE
556	4182595	2-OXO-3-SULFANYLPROPANOATE
557	4369188	ALPHA-CAROTENE
558	4414569	1,2,5-TRIMETHYL-3-[(4-NITROBENZYL)OXY]BENZENE
559	4643300	DIHYDROXYACETONE-P
560	5229060	AC1NQUSA
561	5280338	UNDECAPRENYL PHOSPHATE
562	5280344	COQH2
563	5280346	UBIQUINONE-2
564	5280353	BILIVERDIN
565	5280374	MENAQUINONE-2
566	5280450	LINOLEIC ACID
567	5280483	PHYLLOQUINONE
568	5280489	BETA-CAROTENE
569	5280500	2-MALEYLACETATE
570	5280516	PRECORRIN 2

571	5280523	(2S)-2-ISOPROPYLMALATE
572	5280533	2-ISOPROPYLMALEATE
573	5280591	PREPHYTOENE DIPHOSPHATE
574	5280642	AC1NQXCN
575	5280650	SOLANESYL DIPHOSPHATE
576	5280651	OCTAPRENYL DIPHOSPHATE
577	5280700	DI-TRANS,POLY-CIS-UNDECAPRENYL DIPHOSPHATE
578	5280709	5-FORMYL-2-HYDROXYHEPTA-2,4-DIENEDIOIC ACID
579	5280784	PHYTOENE
580	5280788	ZETA-CAROTENE
581	5280789	NEUROSPORENE
582	5280791	GAMMA-CAROTENE
583	5280801	2-HYDROXYHEPTA-2,4-DIENEDIOATE
584	5280811	PRECORRIN 3A
585	5280829	GERANYLPHENOL
586	5280831	4-HYDROXY-3-OCTAPRENYLBENZOIC ACID
587	5280832	2-OCTAPRENYLPHENOL
588	5280833	3-OCTAPRENYLCATECHOL
589	5280834	2-OCTAPRENYL-6-METHOXYPHENOL
590	5280835	6-METHOXY-2-ALL-TRANS-OCTAPRENYL-1,4-BENZOQUINONE
591	5280836	3-METHYL-6-METHOXY-2-OCTAPRENYL-1,4-BENZOQUINONE
592	5280838	AC1NQXOD
593	5280839	MENAQUINOL
594	5280844	4-HYDROXY-3-POLYPRENYLBENZOATE
595	5280899	ZEAXANTHIN
596	5280916	PRECORRIN 6B
597	5280917	PRECORRIN 6A
598	5280929	PRECORRIN 3B
599	5280930	PRECORRIN 4
600	5280932	PRECORRIN 5
601	5280933	GAMMA-LINOLENIC ACID
602	5280943	AC1NQXSS
603	5281230	DELTA-CAROTENE
604	5281234	ZEINOXANTHIN
605	5281235	BETA-CRYPTOXANTHIN
606	5281909	TRANS,TRANS,CIS-GERANYLGERANYL DIPHOSPHATE

607	5281938	C11542
608	5281939	C11543
609	5281976	HMBPP
610	5281992	METHANOPHENAZINE
611	5281993	DIHYDROMETHANOPHENAZINE
612	5282748	CIS-9-HEPTADECENOIC ACID
613	5283548	DITRANS,OCTACIS-UNDECAPRENYL PHOSPHATE
614	5284607	VITAMIN K1
615	5287432	SHCHC
616	5288700	L-MYO-INOSITOL-1-PHOSPHATE
617	5289105	(R)-PANTOATE
618	5311498	VITAMIN B12
619	5359597	SUPEROXIDE
620	5360545	SODIUM
621	5375177	PLASTOQUINONE
622	5459794	D-MALATE
623	5459820	XYLULOSE 5-PHOSPHATE
624	5459954	IMIDAZOLE GLYCEROL PHOSPHATE
625	5459960	2-HYDROXY-4-OXOPENTANEDIOATE
626	5460048	TRIFORMIN
627	5460228	(S)-2,3-DIHYDRODIPICOLINATE
628	5460271	5-DEHYDROQUINATE
629	5460331	COM-S-S-COB
630	5460360	3-DEHYDROSHIKIMIC ACID
631	5460362	D-ALANYL-D-ALANINE
632	5460413	(6S)-TETRAHYDROFOLIC ACID
633	5460580	ISOCHORISMATE
634	5462148	2-HYDROXY-3-KETO-5-METHYLTHIOPENTENYL-1-PHOSPHATE
635	5462224	MAGNESIUM
636	5462259	(2S)-2-ISOPROPYL-3-OXOSUCCINATE
637	5462261	3-ISOPROPYLMALATE
638	5462265	COENZYME B
639	5462266	5'-PHOSPHORIBOSYLFORMYLGLYCINAMIDINE
640	5462303	(S)-METHYLMALONATE SEMIALDEHYDE
641	5462311	BORON
642	5490066	UDP-MURNAC-ALA

643	5496796	UDP-N-ACETYLMURAMOYL-L-ALANINE
644	6026790	DIACYLGLYCEROL
645	6323531	CHEBI:84805
646	6326742	(6S)-10-HCO-H4FOLATE
647	6326970	SELENIUM
648	6326983	SELENOCYSTEINE
649	6367216	METHYL SELENOL
650	6398465	PROTOPORPHYRIN IX CONTAINING MG
651	6419702	AC1O4WBI
652	6419735	3-HYDROXY-3-METHYL-2-OXOBUTANOATE
653	6433159	LUTEINE
654	6436722	PHYTOFLUENE
655	6437549	EINECS 270-994-0
656	6438349	PHYCOCYANOBILIN
657	6449797	PRESQUALENE DIPHOSPHATE
658	6508102	DIMETHYLMESACONSAURE
659	6540288	DECANETHIOIC ACID
660	6560146	3UWL
661	6857401	(3S)-3-METHYL-2-OXOPENTANOATE
662	6857402	(2R,3S)-3-ISOPROPYLMALATE
663	6857408	AC1OAGKH
664	6857447	BETA-TOCOPHEROL
665	6971070	P-HYDROXYPHENYLPYRUVATE
666	6992112	CHEBI:57822
667	7048523	5-AMMONIO-4-OXOPENTANOATE
668	7048686	(2R)-2,3-DIHYDROXYPROPYL PHOSPHATE
669	7140378	RIBOSYLHYPOXANTHINE MONOPHOSPHATE
670	9543238	4-OXOBUTANOATE
671	9548599	PHOSPHORIBOSYL-FORMIMINO-AICAR-PHOSPHATE
672	9548600	N-(5-PHOSPHO-BETA-D-RIBOSYL)ANTHRANILATE
673	9828112	2-DEOXY-D-RIBOFURANOSE
674	9963391	15-CIS-PHYTOENE
675	9984420	ISORENIERATENE
676	10098570	CHLOROBACTENE
677	10098571	BETA-ISORENIERATENE
678	10218254	BIWLELKAFXRPDE-BUUNGVGGSA-N

679	10219885	PLASTOQUINONE-1
680	10425706	LECITHIN
681	10918539	PROLYCOPENE
682	10918995	GDP-BETA-L-FUCOSE
683	10942192	C04702
684	10946654	2-POLYPRENYL-3-METHYL-5-HYDROXY-6-METHOXY-1,4-BENZOQUINONE
685	10953804	N-ACETYL-ORNITHINE
686	11006912	UDP-N-ACETYLMURAMIC ACID
687	11050836	PHOSPHORIBOSYL-ATP
688	11551129	SELENOSUGAR B
689	11953880	HYDROGENOBYRINATE
690	11953881	PRECORRIN 8
691	11953882	COB(II)YRINATE DIAMIDE
692	11953957	COBALT-PRECORRIN-8
693	11953965	DIVINYLCHLOROPHYLL A
694	11953967	ZINC BACTERIOCHLOROPHYLL A
695	11954010	DEMETHYLPHYLLOQUINONE
696	11954200	PRECORRIN 1
697	11966146	(R)-3-HYDROXYBUTANOYL-COA
698	11966204	TRANS-HEX-2-ENOYL-COA
699	11970143	LIPOPOLYSACCHARIDE
700	11988266	S-METHYL-5-THIO-D-RIBOSE 1-PHOSPHATE
701	11988267	PHOSPHORIBOSYL-AMP
702	12085802	CHLOROPHYLL A
703	12347903	ANTHRACENE-9,10-DIHYDRODIOL
704	13296868	3-FORMYLOXY-PROPANE-1,2-DIOL
705	13831140	LIPID A DISACCHARIDE
706	13999770	(2S)-2-HYDROXY-2-METHYL-3-OXOBUTANOATE
707	14035695	RIBOSE 1,5-BISPHOSPHATE
708	14080393	REDUCED RIBOFLAVIN
709	14506801	5-O-(1-CARBOXYVINYL)-3-PHOSPHOSHIKIMATE
710	15938967	PPGPP
711	15938972	COENZYME II
712	15983957	3-METHYLBUT-3-ENYL DIPHOSPHATE
713	16059194	UDP-N-ACETYLMURAMOYL-L-ALANYL-D-GAMMA-GLUTAMYL-6-CARBOXY-L-LYSYL-D-ALANYL-D-ALANINE

714	16061579	CYCLIC PYRANOPTERIN MONOPHOSPHATE
715	16069658	UDP-GLCNAC
716	16219419	GDP-MAN
717	16220076	UDP-GLCA
718	16667373	UDP-N-ACETYL-ALPHA-D-GLUCOSAMINE
719	16722111	5-METHYLTETRAHYDROPTEROYLTRI-L-GLUTAMATE
720	16722112	TETRAHYDROPTEROYLTRI-L-GLUTAMATE
721	16738692	PHOSPHATIDYLINOSITOL
722	16755619	2-KETOPANTOATE
723	16757114	DTDP-4-AMINO-4,6-DIDEOXY-ALPHA-D-GLUCOSE
724	18666812	5-AMINO-6-(5-PHOSPHO-D-RIBITYLAMINO)URACIL
725	19379894	GERANYL-DIPHOSPHATE
726	20112020	2-[[(2R)-2-AZANYLPROPANOYL]AMINO]PROPANOIC ACID
727	20545621	ACETYLTHIOACETIC ACID
728	21124327	DTDP
729	21145142	(3R)-3-HYDROXY-2-OXO-4-PHOSPHONOOXYBUTANOATE
730	21387441	HHBDDEZLXDKDHK-UHFFFAOYSA-N
731	21604865	3BXG
732	21605869	ME-2,4CPP
733	21933884	2,3-DIHYDROXYISOVALERATE
734	22833559	7-(DIHYDROGEN PHOSPHATE) SEDOHEPTULOSE
735	23657851	5-CARBOXYAMINO-1-(5-PHOSPHO-D-RIBOSYL)IMIDAZOLE
736	23657884	DVTHXBBSXBNVCD-QMUWONGRSA-M
737	23668193	SODIUM NITRITE
738	23724458	UDP-GALACTOSE
739	23724459	UDP-D-XYLOSE
740	23724461	UDP-N-ACETYL-D-GALACTOSAMINE
741	23724478	KOJCFMYSTWNMQW-TZECUDMMSA-N
742	23724479	UDP-3-O-(BETA-HYDROXYMYRISTOYL)-N-ACETYLGLUCOSAMINE
743	23724496	COB(I)YRINATE A,C DIAMIDE
744	23724497	ADENOSYL COBYRINATE A,C DIAMIDE
745	23724498	C06507
746	23724516	COBALT-PRECORRIN 4
747	23724601	6-PHYTYLTOLUQUINOL
748	23724609	(2'S)-DEOXYMYXOL 2'-(2,4-DI-O-METHYL-ALPHA-L-FUCOSIDE)
749	23724610	(3R,2'S)-MYXOL 2'-(2,4-DI-O-METHYL-ALPHA-L-FUCOSIDE)

750	23724612	UNII-2D9J09M91C
751	23724630	THERMOCRYPTOXANTHIN
752	23724631	THERMOZEAXANTHIN
753	23724632	THERMOBISZEAXANTHIN
754	23724672	LIPOYL-AMP
755	23724673	C16242
756	23724675	C16243
757	24755586	9,15,9'-TRI-CIS-ZETA-CAROTENE
758	24762165	S-ADENOSYL-L-METHIONINE
759	24771767	DIHYDRONEOPTERIN PHOSPHATE
760	24771813	D-LYS(UDP-MURNAC-L-ALA-D-GLU)
761	24772978	UDP-N-ACETYL-ALPHA-D-MURAMATE
762	24798720	MURNAC 6-PHOSPHATE
763	24883454	SEPHCHC
764	24892716	COBAMAMIDE
765	24892729	PLASTOQUINOL-1
766	24892737	DTDP-L-DIHYDROSTREPTOSE
767	24906332	S-ACETYLDIHYDROLIPOAMIDE-E
768	25200933	UDP-SULFOQUINOVOSE
769	25201749	UDP-3-O-(BETA-HYDROXYMYRISTOYL)-D-GLUCOSAMINE
770	25202390	DTDP-ALPHA-D-GLUCOSE
771	25202391	(S)-2-AMINO-6-OXOPIMELATE
772	25202832	4-AMINO-5H-IMIDAZOLE-5-CARBOXAMIDE
773	25203490	DECARBOXYLATED SAM
774	25203769	UDP-2,3-BIS(3-HYDROXYTETRADECANOYL)GLUCOSAMINE
775	25243937	CHEBI:57776
776	25243979	GDP-4-DEHYDRO-ALPHA-D-RHAMNOSE
777	25243985	UDP-N-ACETYL-ALPHA-D-MURAMOYL-L-ALANYL-GAMMA-D-GLUTAMYL-MESO-DIAMINOPIMELATE
778	25244236	ALPHA-D-MANNOPYRANOSE 6-PHOSPHATE
779	25244331	2,5-DIAMINO-6-(1-D-RIBOSYLAMINO)PYRIMIDIN-4(3H)-ONE 5'-PHOSPHATE
780	25244516	IMIDAZOLE ACETOL-P
781	25244603	SOLANYL PYROPHOSPHATE
782	25244751	PRONEUROSPORENE
783	25244831	PHYTYL DIPHOSPHATE(3-)
784	25244866	LIPID A DISACCHARIDE

785	25244881	UDP-GLCNAC-ENOLPYRUVATE
786	25245190	URIDINE DIPHOSPHATE N-ACETYLMANNOSAMINE
787	25245199	5-AMINO-6-(5-PHOSPHO-D-RIBOSYLAMINO)URACIL
788	25245232	CHEBI:58380
789	25245380	[5-[(2-FORMAMIDOACETYL)AMINO]-3,4-DIHYDROXY-TETRAHYDROFURAN-2-YL]METHYL PHOSPHATE
790	25245438	3-ACETYLOXY-2-AZANIUMYLPROPANOATE
791	25245473	STREPTOMYCIN 6-PHOSPHATE(1+)
792	25245548	3-PHOSPHO-D-GLYCERIC ACID
793	25245607	MANNOSE-1-PHOSPHATE
794	25245609	4-P-N-PANTOTHENOYLCYSTEINE
795	25245613	PRECORRIN-3A
796	25245635	DI-TRANS,OCTA-CIS-UNDECAPRENYL PHOSPHATE
797	25245653	UNDECAPRENYL PHOSPHATE(2-)
798	25245982	CHEBI:57510
799	25246209	UDP-2,3-BIS(3-HYDROXYMYRISTOYL)GLUCOSAMINE
800	35028442	5(Z), 14(Z)-EICOSADIENOIC ACID
801	36688186	6-PHOSPHONATOOXY-D-GLUCONATE
802	42626431	(6S)-5-METHYLTETRAHYDROFOLATE
803	42626647	PZWJECBEQGYZFR-CROAGEEISA-N
804	44140569	1,4-DIHYDROXY-2-NAPHTHOYL-COA
805	44147587	1,2-DISTEAROYL PHOSPHATIDYL SERINE
806	44228982	4'-PHOSPHO-D-PANTETHEINE
807	44229200	MAGNESIUM PROTOPORPHYRIN MONOMETHYL ESTER
808	44229246	131-HYDROXY-MG-PROTOPORPHYRIN IX 13-MONOMETHYL ESTER
809	44237185	MSBQ
810	44602414	ATNHDLDRLWWWCB-AENOIHSZSA-M
811	46173549	PPPGPP
812	46173708	COB(I)ALAMIN
813	46173713	N-ACETYL-ALPHA-D-GLUCOSAMINYL-1-DIPHOSPHO-DITRANS,POLYCIS-UNDECAPRENOL
814	46173745	COBINAMIDE
815	46173749	CHEBI:27692
816	46173753	C05898
817	46173758	C06509
818	46173760	ADENOSYLCOBINAMIDE-GDP

819	46173804	(6R)-6-(L-ERYTHRO-1,2-DIHYDROXYPROPYL)-5,6,7,8-TETRAHYDRO-4A-HYDROXYPTERIN
820	46878371	(6R)-5,10-METHENYLTETRAHYDROFOLATE
821	46878406	O-PHOSPHO-L-HOMOSERINE
822	46926108	CHLOROPHYLL B
823	51351778	15,9'-DI-CIS-PHYTOFLUENE
824	53323638	CHEBI:67205
825	54675765	5-CARBOXYMETHYL-2-HYDROXYMUCONATE
826	56928068	C05172
827	56928124	DEMETHYLMENAQUINOL
828	56928127	C19859
829	71448891	O-SUCCINYLBENZOYL-COA
830	71448907	C16244
831	71768102	2,3-DIMETHYL-5-PHYTYLQUINOL
832	71768135	2-METHYL-6-PHYTYLQUINOL
833	124222233	LJYQKLDIBPYLED-FWQOJXOKSA-M

*CID, compound identification number by PubChem database
