# Supplementary material for: Network analysis retrieving bioactive compounds from Spirulina (Arthrospira platensis) and their targets related to systemic lupus erythematosus
Source: PLoS One. 2024 Aug 29;19(8):e0309303. doi: 10.1371/journal.pone.0309303 (PMC11361558; doi:10.1371/journal.pone.0309303)
Supplement: S4 Table — (PDF) [file pone.0309303.s005.pdf]

S4 Table. List of 281 agents/drugs with known immunosuppressive or immunomodulating activities, along with their corresponding compound identifier numbers in the PubChem database.

No	CID*	Immunosuppressive or immunomodulatory drugs/agents
1	237	QUINACRINE
2	320	NSC277817
3	596	BETA-ARABINOSYLCYTOSINE
4	643	DESTHIOBIOTIN
5	2241	ASCOMYCIN
6	2244	ASPIRIN
7	2265	AZATHIOPRINE
8	2478	BUSULFAN
9	2719	CHLOROQUINE
10	2901	CYCLOLEUCINE
11	2907	CYCLOPHOSPHAMIDE
12	2909	CYCLOSPORIN A
13	3108	DIPYRIDAMOLE
14	3206	9-(2-HYDROXY-3-NONYL) ADENINE
15	3385	5-FLUOROURACIL
16	3476	GLIMEPIRIDE
17	3652	HYDROXYCHLOROQUINE
18	3690	IFOSFAMIDE
19	3899	LEFLUNOMIDE
20	3946	LOBENZARIT
21	4112	AMETHOPTERIN
22	4247	DANSYLCADAVERINE
23	4739	VIDARBINE
24	5050	REPIRINAST
25	5372	FK-506
26	5422	ISOTETRANDRINE
27	5426	THALIDOMIDE
28	5433	RACEFENICOL
29	5743	DEXAMETHASONE
30	5755	PREDNISOLONE
31	5865	PREDNISONE
32	5881	DEHYDROEPIANDROSTERONE

33	6223	GLIOTOXIN
34	6252	CYTARABINE HYDROCHLORIDE
35	6253	CYTARABINE
36	6436	TRIAMCINOLONE ACETONIDE
37	6497	DIMETHYL SULFATE
38	6730	BIS(4-CHLOROPHENYL) ACETIC ACID
39	6741	METHYLPREDNISOLONE
40	8019	2-METHOXYETHANOL
41	10341	2(5H)-FURANONE
42	12251	METHOXYACETIC ACID
43	12688	DIBUTYLTIN DICHLORIDE
44	14403	AFBI
45	15979	PRISTANE
46	18343	DOXIFLURIDINE
47	20279	CLADRIBINE
48	22420	CYCLOPHOSPHAMIDE MONOHYDRATE
49	25447	COFORMYCIN
50	27200	THIAMPHENICOL
51	30623	RAZOXANE
52	30751	FLUDARABINE PHOSPHATE
53	31269	CARBAMOYLIMINOUREA
54	31593	BENZNIDAZOLE
55	40632	PIRFENIDONE
56	40926	CO-V; VIDARBINE
57	47462	LOBENZARIT DISODIUM
58	50587	THYMOPOIETIN PENTAPEPTIDE
59	54120	STEPRONIN
60	54343	DALTROBAN
61	54445	CASTANOSPERMINE
62	55361	GUSPERIMUS TRIHYDROCHLORIDE
63	55362	GUSPERIMUS
64	55390	PIRAMAGREL
65	57030	BREQUINAR
66	57782	(+/-)-LISOFYLLINE
67	59757	TEPOXALIN
68	60749	GEMCITABINE HYDROCHLORIDE

69	60750	GEMCITABINE
70	62280	CYCLOSPORINE
71	64988	PMEDAP
72	65370	GLATIRAMER
73	65384	L-GLUTAMIC ACID-L-TYROSINE COPOLYMER
74	65702	TROFOSFAMIDE
75	68784	TRAXANOX
76	73078	TETRANDRINE
77	83273	NITROSODIMETHYLUREA
78	91271	UNII-S0D7V00ZRX
79	96849	8-AMINOGUANOSINE
80	104762	MIZORIBINE
81	104895	CP55940
82	104933	TRILAZAD MESYLATE
83	104934	U-74389G
84	107793	SAIKOSAPONIN D
85	107964	UNII-IDW4RCM01L
86	107969	FINGOLIMOD HYDROCHLORIDE
87	107970	FINGOLIMOD
88	107985	TRIPTOLIDE
89	108037	2-ATHBI
90	114682	ARABINOFURANOSYLCYTOSINE
91	114844	LEU-LEU-OME
92	114917	TANSHINONE I
93	121750	WR99210
94	121957	SKF 105685
95	122000	CONTRAGESTAZOL
96	122651	DIDEMNIN B
97	123844	DIDEMNIN A
98	126941	METHOTREXATE
99	134780	POMALIDOMIDE
100	140765	2(3H)-FURANONE
101	149784	(2R,3S)-3-(6-AMINOPURIN-9-YL) NONAN-2-OL
102	149793	(2S,3S)-3-(6-AMINOPURIN-9-YL) NONAN-2-OL
103	161333	METHOXYACETATE
104	164676	TANSHINONE IIA

105	165528	METHOTREXATE HYDRATE
106	167928	SAIKOSAPONIN A
107	176872	HKVAMNSJSFKALM-DHGBBOBXSA-N
108	186907	AFLATOXIN B1
109	189821	DEFLAZACORT
110	216326	LENALIDOMIDE
111	259808	2,8-DIAMINO-9-[3,4-DIHYDROXY-5-(HYDROXYMETHYL)-2-OXOLANYL]-3H-PURIN-6-ONE
112	298076	RAZOXANE, (R)-
113	312145	WORTMANNIN
114	318797	TANSHINONE II-B
115	433294	LITHIUM CHLORIDE
116	439693	PENTOSTATIN
117	445027	D-DETHIOBIOTIN
118	445643	TACROLIMUS
119	451417	THYMOPENTIN
120	457825	ISOTETRANDRINE
121	460129	ZINC3977741
122	478951	AC1L9ZMV
123	501254	(R)-LISOFYLLINE
124	550898	DIDEMNIN B
125	637568	DIMETHYL FUMARATE
126	667490	6-MERCAPTOPURINE
127	676166	6-MERCAPTOPURINE RIBOSIDE
128	2724350	6-MERCAPTOPURINE MONOHYDRATE
129	2861016	NSC406021
130	3055172	PIMECROLIMUS
131	3081884	GLATIRAMER ACETATE
132	4412255	5-(1,1-DIMETHYLHEPTYL)-2-[5-HYDROXY-2-(3-HYDROXYPROPYL) CYCLOHEXYL] PHENOL
133	5280754	CYCLOSPORIN A
134	5281051	HYPERICIN
135	5281078	MYCOPHENOLATE MOFETIL
136	5281800	ACTEOSIDE
137	5282071	ASCOMYCIN
138	5282315	TACROLIMUS HYDRATE
139	5284344	AZASERINE

140	5284373	CYCLOSPORIN A
141	5284616	RAPAMYCIN
142	5287543	AC1NR9VL
143	5288149	SEOCALCITOL
144	5288670	LEXACALCITOL
145	5311219	ENISOPROST
146	5321894	L-TETRANDRINE
147	5351180	CYTARABINE HYDROCHLORIDE
148	5351212	ISOTETRANDRINE
149	5353425	NSC166613
150	5358081	NSC226080
151	5374464	ANTIBIOTIC AY 22989
152	5458585	CYCLOSPORIN A
153	5458707	DIDEMNIN B
154	5458826	DEOXYSPERGUALIN
155	5460439	RAPAMYCIN
156	5462814	AZODICARBONAMIDE
157	5475158	CINANSERIN
158	5497196	RAPAMYCIN (TN)
159	5745214	SEMAPIMOD
160	6433141	CINANSERIN HYDROCHLORIDE
161	6435893	CYCLOSPORINE
162	6436030	QFJCIRLUMZQUOT-WMVIXHMVSA-N
163	6436101	GECLOSPORIN [INN]
164	6437370	ASCOMYCIN
165	6438358	ONO-4057
166	6438379	UNDECYLPRODIGIOSIN
167	6438394	MYRIOCIN
168	6441022	MYCOPHENOLATE MOFETIL HYDROCHLORIDE
169	6442177	EVEROLIMUS
170	6447131	PIMECROLIMUS
171	6475296	CYCLOSPORIN G
172	6475629	ASCOMYCIN
173	6507974	ASCOMYCIN
174	6509979	PIMECROLIMUS
175	6711227	DIDEMNIN B

176	6917688	ATRIMUSTINE
177	6918251	OFATUMUMAB
178	6918289	TEMSIROLIMUS
179	6918486	VOCLOSPORIN
180	9549174	ASCOMYCIN
181	9554199	SEMAPIMOD TETRAHYDROCHLORIDE
182	9854380	QFJCIRLUMZQUOT-LBFZIKHJSA-N
183	9855081	CYCLOSPORIN
184	9875547	SAIKOSAPONIN B1
185	9876378	ZOTAROLIMUS
186	9896684	U-74389G
187	9926694	TANSHINONE IIB
188	9926791	TOFACITINIB
189	9963211	DIDEMNIN C
190	10011321	TTZALNKZCLGFGS-PUUGUDBISA-N
191	11056106	EHNA
192	11158972	UMIROLIMUS
193	11329481	METHOTREXATE DISODIUM SALT
194	11520894	DEFOROLIMUS
195	11529527	AZATHIOPRINE SODIUM
196	11561674	APREMILAST
197	11671467	FOSTAMATINIB
198	11953994	UNDECYLPRODIGIOSIN
199	11957547	EHNA HYDROCHLORIDE
200	11969572	77327-04-9
201	13179970	TRAXANOX SODIUM PENTAHYDRATE
202	13895197	DIDEMNIN
203	14608008	5'-DEOXY-5'-FLUOROURIDINE
204	16051947	PIMECROLIMUS
205	16197723	UNII-4TWN6SZB8W
206	16197725	ALICAFORSEN
207	16211121	HKVAMNSJSFKALM-AJDIITERSA-N
208	16682746	TRIBUTYLTIN OXIDE
209	16760631	QFJCIRLUMZQUOT-MTKXUJSSSA-N
210	17753757	PIMECROLIMUS
211	20055510	EB-1089

212	21120116	KQKVCZQOVVBOEE-UHFFFAOYSA-N
213	21637642	SAIKOSAPONIN B2
214	23223059	BIPHENQUINATE
215	23663399	CLEARNAL
216	23663964	BREQUINAR SODIUM
217	23665584	MYCOPHENOLATE SODIUM
218	23678403	AC1Q1V2K
219	23678981	METHOTREXATE SODIUM
220	23691748	BRONCOPLUS
221	23724849	AFINITOR (TN)
222	24178294	MONO-[123I] IODOHYPERICIN
223	24197375	EHNA HYDROCHLORIDE
224	24801580	RITUXAN (TN)
225	24838593	DEOXYSPERGUALIN
226	24871327	NCGC00181331-01
227	24883466	CYCLOSPORINE
228	24906323	METHOTREXATE SODIUM
229	24916955	6-EPICASTANOSPERMINE
230	25244917	DESTHIOBIOTIN
231	25246316	CYCLOSPORINE
232	44201989	MLS002473167
233	44202892	SAIKOSAPONIN A
234	44202893	SAIKOSAPONIN B
235	44326589	YOOVTUPUBVHMPG-JOAPDPHISA-N
236	45280821	DIDEMNIN B
237	46173839	DIDEMNIN B
238	46930999	EVEROLIMUS (RAD001)
239	49867938	CYCLOSPORIN A
240	53394893	PIMECROLIMUS
241	53398658	AFINITOR (R)
242	53482953	QFJCIRLUMZQUOT-PBQOLAGSSA-N
243	53486290	PIMECROLIMUS
244	54600319	ANTIBIOTIC AY 22989
245	54608520	42-O-(2-HYDROXYETHYL) RAPAMYCIN
246	54684141	TERIFLUNOMIDE
247	56603701	D03MTN

248	60148419	PHARMAKON1600-01504008
249	60150249	RAPAMYCIN (SIROLIMUS)
250	60150250	HKVAMNSJSFKALM-DHGBBOBXSA-N
251	69621045	CYTARABINE HYDROCHLORIDE
252	70789204	HKVAMNSJSFKALM-DHGBBOBXSA-N
253	71296105	HKVAMNSJSFKALM-DHGBBOBXSA-N
254	71296106	QFJCIRLUMZQUOT-MHOVTDRTSA-N
255	71306795	CYCLOSPORIN A
256	71308609	RAPAMYCIN
257	71433934	U-74389G
258	71463825	CYCLOSPORINE A
259	71627180	AZASERINUM
260	73013349	THYMOPENTINE
261	73417192	4Z8R6ORS6L
262	73417324	NTIXXMPXAKYDDN-XJCLVHIMSA-N
263	73896891	DYCLOSPORIN A
264	73946544	KLZOTDOJMRMLDX-KKWMEHBLSA-N
265	76957788	SXJYLNBCGUJKNY-MTPWEOJCSA-N
266	76967668	TZLQTBZXPPCQCC-IJHYXJKASA-N
267	91885489	AOB6822
268	91933106	HKVAMNSJSFKALM-DHGBBOBXSA-N
269	98042555	AKOS026750140
270	99719685	QFJCIRLUMZQUOT-OKUYTRSTSA-N
271	100926147	HKVAMNSJSFKALM-DHGBBOBXSA-N
272	100962152	SAIKOSAPONIN K
273	101280122	UNDECYLPRODIGIOSIN
274	101594417	77327-04-9
275	101598330	DIDEMNIN B
276	101927486	TTZALNKZCLGFGS-RAKFRCPGSA-N
277	102572331	PIMECROLIMUS
278	102602666	ASCOMYCIN
279	102602669	HKVAMNSJSFKALM-DHGBBOBXSA-N
280	118701616	ASCOMYCIN
281	118701644	RAPAMYCIN (TN)

*CID, compound identifier number by PubChem database
