# Supplementary material for: Network analysis retrieving bioactive compounds from Spirulina (Arthrospira platensis) and their targets related to systemic lupus erythematosus
Source: PLoS One. 2024 Aug 29;19(8):e0309303. doi: 10.1371/journal.pone.0309303 (PMC11361558; doi:10.1371/journal.pone.0309303)
Supplement: S5 Table — (PDF) [file pone.0309303.s006.pdf]

S5 Table. Examples and frequency of structural similarity matchings between 833 compounds retrieved from *A. platensis* C1 and 281 immunosuppressive agents with high Tanimoto scores ranging from 60 to 100%.

Tanimoto score	Number of matchings	Example of the matchings	
		Compounds	Immunosuppressive agents
100%	4	-Cytidine<br>-Desthiobiotin<br>-Agmatine<br>-Anthranilic acid	-Beta-arabinosylcytosine<br>-Cytarabine<br>-Arabinofuranosylcytosine (Iretin)<br>-D-dethiobiotin<br>-Agmatine<br>-Anthranilic acid
$\geq$ 95%	28	-Adenylylselenate<br>-Deoxyadenosine diphosphate<br>-Adenosine<br>-Adenylyl sulfate<br>-cAMP	-Cytarabine hydrochloride<br>-Destiobiotin<br>-Fludarabine phosphate<br>-Cladribine
$\geq$ 90%	103	-Acetyl adenylate<br>-Xanthosine<br>-Uridine	-5’-Deoxy-5’-fluorouridine<br>-L-glutamic acid<br>-Gembitabine

		-L-tyrosine<br><br>-Adpribose	
$\geq$ 85%	203	-Decarboxylated SAM<br><br>-Imidazole glycerol phosphate<br><br>-Adenylosuccinic acid<br><br>-5'-Xanthylic acid<br><br>-Xanthosine monophosphate	-Metrotrexate<br><br>-Glatiramer acetate<br><br>-6-Mercaptopurine riboside<br><br>-Mizoribine<br><br>-5-Fluorouracil
$\geq$ 80%	412	-Denethylmenaquinol<br><br>-Lutein<br><br>-Biotin<br><br>-Thymine<br><br>-Anthranilic acid	-Seocalcitol<br><br>-Doxifluridine<br><br>-Lisofylline<br><br>-2-methoxyacetic acid<br><br>-Lobenzarit
$\geq$ 60%	4,447	-Cobinamide<br><br>-Precorrin-3A<br><br>-4-Aminobenzoic acid<br><br>-1-(2-carboxyphenylamino)-1-<br><br>deoxyribulose<br><br>5-phosphate<br><br>-Phycocyanobilin	-Rapamycin<br><br>-Ascomycin<br><br>-Cyclosporin A<br><br>-Lobenzarit<br><br>-Semapimod
