# Supplementary material for: Network analysis retrieving bioactive compounds from Spirulina (Arthrospira platensis) and their targets related to systemic lupus erythematosus
Source: PLoS One. 2024 Aug 29;19(8):e0309303. doi: 10.1371/journal.pone.0309303 (PMC11361558; doi:10.1371/journal.pone.0309303)
Supplement: S6 Table — (PDF) [file pone.0309303.s007.pdf]

S6 Table. List of 660 bioassay identifier numbers associated with systemic lupus erythematosus, retrieved from the PubChem database.

538	145184	277225	392775	566829	731559
581	145318	277226	392776	566830	731560
606	145325	277227	392777	568976	731561
640	145328	277264	392778	575658	731562
697	145330	277275	392779	575810	731563
787	146070	279734	392780	575816	731564
829	146076	280088	431209	575873	731565
832	146080	280089	435024	578660	731566
1031	146213	280090	435027	578767	731567
1253	156811	281264	443579	588336	731568
1338	156812	281267	443580	588339	731569
1779	156813	281276	443581	588340	731570
1784	156814	282079	443582	588341	731571
1788	165929	290973	443583	590108	731572
1793	165930	290974	443584	590109	731573
1852	165931	292389	444582	593976	731574
1904	165932	292616	444583	598078	731575
1921	165933	292617	444584	598080	731576
1924	165934	298248	444589	598668	731577
2126	165935	298256	444590	602351	731578
2135	196564	298257	444733	602353	731579
2140	204507	298262	444736	606299	731580
2337	210150	301330	449726	609337	731581
2483	215455	303936	449727	609338	732098
2485	215456	307331	450691	609342	732101
2682	215457	307333	453980	609344	732102
2686	215589	307445	453981	609345	732615
2801	222297	307754	453982	609346	732618
2956	222299	307755	453983	609347	732619
2957	222300	309309	453984	622616	732628
7575	222301	309317	453985	624099	732923
23980	226363	311018	454785	624355	732924
23981	226364	316534	458538	624406	733374
48199	226365	316535	461440	625175	733375

48219	226499	316571	464954	625176	733376
48221	233468	316582	466963	642291	733377
66475	233469	316636	468995	642292	733378
66492	233470	316637	470233	642293	733379
66495	238592	320726	470234	644885	733380
66638	239119	320728	478473	644886	733381
66671	239395	320731	478474	644889	733382
66813	239518	320977	478638	644890	733383
66830	239664	321181	478639	651757	733384
66836	239665	326197	480453	651810	736485
66839	241156	330855	480454	651811	739718
66974	241241	330857	480455	654953	739719
66987	241242	331095	480456	654954	739722
67010	241909	331096	480457	654955	739723
67013	242594	331097	480458	654956	742485
67161	242695	331098	480459	654970	742486
67482	242732	331099	480460	654971	742576
67484	243346	331100	480461	662771	742577
67485	243358	331101	480462	664987	742583
67493	243374	331102	488869	664988	742688
67494	243429	331103	488871	664989	742867
73794	243430	331104	488884	664990	743121
89096	243431	331105	490653	664991	743454
91007	243432	331106	490654	664992	743458
99435	243433	331107	500171	664993	743961
99451	244096	331108	500173	664994	743962
99579	244246	331109	504189	664995	743964
101346	244255	331110	504191	664996	748039
143006	247750	331111	504734	664997	750022
143070	254383	331112	504750	664998	751802
143079	255254	331113	507742	664999	751803
143152	255257	331114	508161	665000	754717
143255	257602	331115	511078	665001	754718
143293	257603	331116	511079	666389	754720
143295	257821	331117	511080	674022	754721
143305	257822	331118	513622	674032	754728

143306	259220	331119	513623	689747	754729
143320	259801	331120	526325	689749	755470
143472	262014	331121	526572	698493	756058
143475	262015	331122	526573	698497	756059
143606	262318	331123	526576	706152	762517
143765	262624	331124	526577	706159	762518
143781	263641	331125	526578	706160	762519
143786	263694	331126	526655	706161	762520
143921	263697	332028	526807	707052	762521
144196	265079	332029	526808	707706	762523
144462	265080	340319	526809	707918	762527
144466	265081	340381	526810	709969	763538
144475	266543	351586	526811	709970	769981
144597	266544	351587	526812	709971	769982
144602	266545	351588	538753	709972	769990
144606	267359	351590	538754	709973	769992
144609	267409	360111	538755	709974	769993
144610	267499	360112	540250	709975	769994
144622	267500	360117	540251	709976	777151
144672	267563	360118	547679	709977	781194
144695	267564	360119	566809	709978	781220
144762	267565	360120	566810	710278	1057640
144765	267566	360121	566811	711549	1058710
144767	269510	360122	566812	720520	1058712
144826	269533	360123	566813	720521	1060258
144830	269534	360125	566814	720529	1060362
144836	269535	360126	566815	720710	1060363
144891	269536	360128	566816	722024	1060810
144895	269537	360129	566817	724894	1060811
144899	269538	360131	566818	724895	1064680
144966	270297	360132	566819	730395	1064681
144973	270544	364620	566820	730403	1064682
144974	270545	364621	566821	731551	1064683
145032	271814	384542	566822	731552	1064684
145033	271828	384543	566823	731553	1064795
145038	272849	384544	566824	731554	1069033

145102	272852	384545	566825	731555	1069034
145108	274172	385700	566826	731556	1069035
145173	274173	385701	566827	731557	1076443
145176	274174	385702	566828	731558	1076450
